# Dynamic effects on ligand field from rapid hydride motion in an iron(ii) dimer with an *S* = 3 ground state†‡

**DOI:** 10.1039/d2sc06412j

**Published:** 2023-02-08

**Authors:** Sean F. McWilliams, Brandon Q. Mercado, K. Cory MacLeod, Majed S. Fataftah, Maxime Tarrago, Xiaoping Wang, Eckhard Bill, Shengfa Ye, Patrick L. Holland

**Affiliations:** a Department of Chemistry, Yale University New Haven Connecticut USA patrick.holland@yale.edu; b Max Planck Institute for Chemical Energy Conversion Mülheim an der Ruhr Germany; c Neutron Sciences Directorate, Oak Ridge National Laboratory Oak Ridge Tennessee USA; d State Key Laboratory of Catalysis, Dalian Institute of Chemical Physics, Chinese Academy of Sciences Dalian China shengfa.ye@dicp.ac.cn

## Abstract

Hydride complexes are important in catalysis and in iron–sulfur enzymes like nitrogenase, but the impact of hydride mobility on local iron spin states has been underexplored. We describe studies of a dimeric diiron(ii) hydride complex using X-ray and neutron crystallography, Mössbauer spectroscopy, magnetism, DFT, and *ab initio* calculations, which give insight into the dynamics and the electronic structure brought about by the hydrides. The two iron sites in the dimer have differing square-planar (intermediate-spin) and tetrahedral (high-spin) iron geometries, which are distinguished only by the hydride positions. These are strongly coupled to give an *S*_total_ = 3 ground state with substantial magnetic anisotropy, and the merits of both localized and delocalized spin models are discussed. The dynamic nature of the sites is dependent on crystal packing, as shown by changes during a phase transformation that occurs near 160 K. The change in dynamics of the hydride motion leads to insight into its influence on the electronic structure. The accumulated data indicate that the two sites can trade geometries by rotating the hydrides, at a rate that is rapid above the phase transition temperature but slow below it. This small movement of the hydrides causes large changes in the ligand field because they are strong-field ligands. This suggests that hydrides could be useful in catalysis not only due to their reactivity, but also due to their ability to rapidly modulate the local electronic structure and spin states at metal sites.

## Abstract 2

Crystallographic, spectroscopic, and computational studies on diiron(ii) hydride complexes reveal rapid hydride motions that cause major changes in the electronic structures of the iron sites.

## Graphical abstract

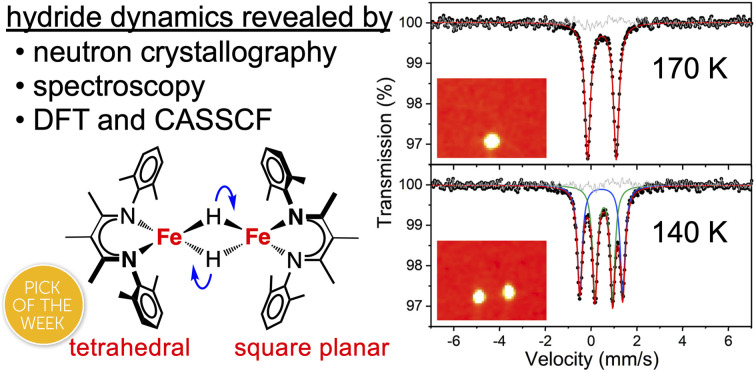

## Introduction

The capabilities of transition-metal hydride complexes in catalysis have made them a favorite target of organometallic chemists. These hydride species are an indelible part of fundamental reactions such as β-hydride elimination, migratory insertion into substrate multiple bonds, C–H activation and H_2_ consumption/production.^1–5^ In addition to these two-electron reactions, hydrides can undergo one-electron reactions; for example, hydrogen atom transfer (HAT) to alkenes generates an alkyl radical that can be trapped to give cross-coupling products with novel selectivites.^6^ Electron transfer reactions of hydride species are also important in natural metalloenzymes such as hydrogenases and nitrogenases,^7,8^ and in electrocatalysts and photocatalysts for H_2_ production,^9^ further emphasizing the importance of one-electron changes of hydride complexes.

Given this broad range of one- and two-electron reactions in diverse coordination environments, the spin states of hydride complexes have been much less studied.^10^ Open-shell hydride complexes are strongly implicated in unobserved catalytic intermediates, for example nitrogenase cofactors (which have iron–sulfur clusters in weak-field environments)^11–14^ and HAT catalysts for alkene hydrofunctionalization (which often have only weak-field acac or salen ligands).^15^ Thus there is strong motivation for understanding the electronic structure of hydride ligands in open-shell systems. However, very few isolated hydride complexes are known that are paramagnetic.^10,16^

In the work described here, we focus on iron(ii), which is found in many of the above catalysts. The conventionally found ground spin states of iron(ii) are low-spin (*S* = 0, often the ground state in octahedral geometry) or high-spin (*S* = 2, often the ground state in tetrahedral or octahedral geometries). An intermediate-spin electronic configuration (*S* = 1) is less commonly the ground state, but this can occur in a square-planar ligand field that raises the energy of the d_*x*^2^–*y*^2^_ orbital enough that it remains unoccupied. A d^6^ system with an intermediate-spin ground state was first observed and explained in (tetraphenylporphyrin)iron(ii).^17,18^ More recently, Chirik *et al.* used a pincer ligand and varying steric demands to explore the balance between square-planar and tetrahedral geometries, and similarly observed *S* = 1 ground states for square-planar iron(ii) and *S* = 2 ground states for tetrahedral iron(ii).^19^ However, a square-planar geometry does not necessarily give an intermediate-spin ground state.^20–25^

Different spin states engender different metal–ligand bond lengths, with bonds being shortest in low-spin iron complexes and longer in open-shell iron complexes because the population of metal–ligand antibonding orbitals in the higher spin states weakens the bonding interactions. However, M–H distances are not known for the few open-shell hydrides, because locating hydrides accurately requires a neutron crystal structure. Correlating these differences with reactivity is also challenging because the spin state can change during a reaction.^26–31^

**Fig. 1:**
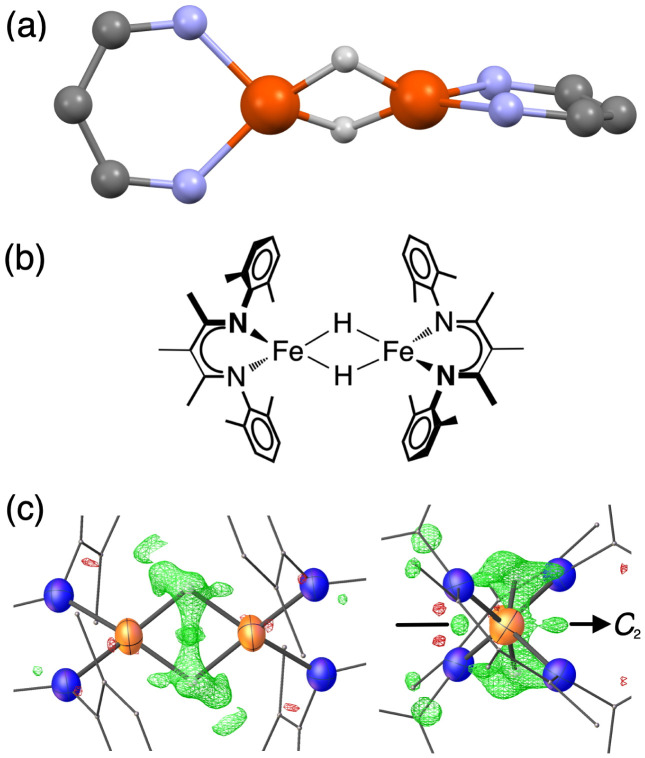
(a) Ball-and-stick model showing the core of the X-ray crystal structure of 1 at 223 K,^35^ emphasizing that the β-diketiminate supporting ligands are perpendicular to one another. The pairs of Fe centers and the pairs of hydrides are rendered crystallographically equivalent by the operator 
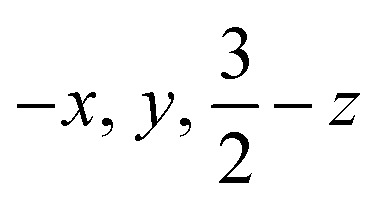
. (b) Chemical structure of 1, showing the composition of the diketiminate ligands. (c) Two views of the previously reported X-ray crystal structure of 1 at 223 K (left: along the 2-fold axis; right: along the Fe–Fe axis), with the Fe and N atoms as thermal ellipsoids. The residual electron density (*F*_obs_–*F*_calc_) is shown as a green wireframe.

In the quest to understand open-shell hydride species, β-diketiminate ligands have been beneficial, because they are modular and tunable weak-field ligands.^32^ We have isolated iron(ii) hydride complexes with three related *N*,*N*′-diaryldiketiminate ligands, and each was crystallographically characterized as a dimer with two bridging hydrides.^33–35^ In related work, Murray used a macrocyclic β-diketiminate to create complexes with a triiron core bridged by three hydrides.^36^ In each β-diketiminate-supported iron hydride complex, Mössbauer and magnetism studies have led the authors to conclude that the ground state has high-spin electronic configurations at the iron sites. This is reasonable since a wide variety of related mononuclear β-diketiminate-supported iron(ii) complexes have *S* = 2 ground states with related σ-donor ligands such as alkyl, aryl, alkoxide, amide, and halide.^37^ However, in this manuscript we reveal that the motion of hydrides in the hydride dimer 1 (Fig. 1) can surprisingly cause the iron(ii) sites to flip between high-spin and intermediate-spin electronic configurations rapidly. The intermolecular interactions, ligand-field changes, and hydride motions that bring about this spin-state change are elucidated using X-ray and neutron crystallography, magnetism, spectroscopy, and computations. These data illustrate that hydride ligands are unique because of their rapid motions and strong ligand field, a combination that has implications for the roles of hydrides in catalysis.

## Results

### Desymmetrization with temperature

Compound 1 (Fig. 1) and its deuterated analogue 1-D were reported previously.^35,38^ In the reported X-ray crystal structure of 1 collected at 223 K (space group *C*2/*c*, unique *b* axis, with *Z* = 4)^35^ there is a 2-fold rotation axis parallel to the crystallographic *b* axis, which passes through each molecule perpendicular to the Fe_2_H_2_ rhomb, and which renders the two halves of the molecule equivalent (Fig. 1c, right). A small peak in the electron density map consistent with the hydride position was observed at a location 1.59(4) Å from the iron position, and has a symmetry-related peak that lies 1.88(4) Å away from this iron atom. However, the positions of hydrides are unreliable from X-ray crystallography, and the *F*_obs_–*F*_calc_ map displays residual electron density near the hydrides (green wireframe in Fig. 1c).

**Fig. 2:**
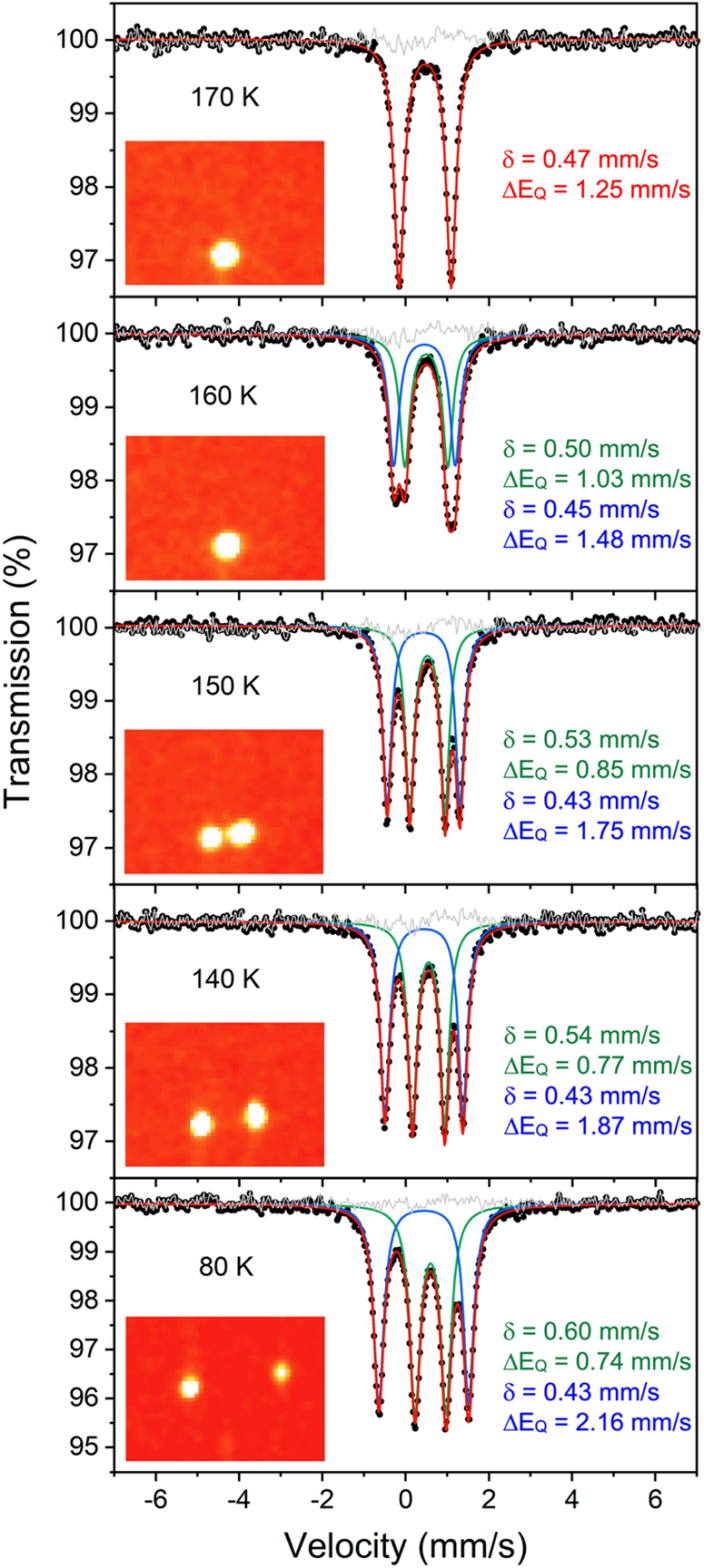
Mössbauer data of crystalline 1 (toluene-free crystal). Each orange inset shows an equivalent section of the diffraction pattern, corresponding to the (−8 −4 6) reflection in the high-temperature indexing in *C*2/*c*, and the splitting of the reflection at low temperature is a result of conversion to the twinned structure. Each main panel shows the Mössbauer data (black circles) with the associated fits (red) that are the sum of components for the tetrahedral site (green) and the square-planar site (blue). The residuals are shown in grey.

Our previous analysis using the refined hydride positions had been that each iron(ii) site has a geometry that lies between square planar and tetrahedral.^35,38^^1^H NMR spectra of 1 show the expected number of peaks for equivalent diketiminate environments, and cooling a sample in toluene-*d*_8_ to 188 K showed no evidence of dynamic behavior. The solution effective magnetic moment at room temperature in C_6_D_6_ is 7.0(4) Bohr magnetons (*μ*_B_) per dimer, which was consistent with the previous assessment that 1 had two equivalent *S* = 2 subsites with little to no exchange coupling (two uncoupled *S* = 2 ions predict a spin-only value of 6.9 *μ*_B_).^39^ This model was supported by the Mössbauer spectrum of a solid at 170 K (Fig. 2, top), which showed a doublet with an isomer shift (*δ*) of 0.48 mm s^−1^ and a quadrupole splitting (Δ*E*_Q_) of 1.25 mm s^−1^, which are similar to those in low-coordinate iron(ii) alkyl complexes that have high-spin ground states.^40^

However, the measurements at lower temperature are inconsistent with equivalent iron sites. Namely, cooling single crystals of 1 below 170 K leads to splitting in the X-ray diffraction data and in the Mössbauer spectrum (Fig. 2). The crystallographic data indicate a change in the unit cell, causing the peaks in the diffraction pattern to gradually split as the temperature is lowered (orange insets in Fig. 2). At 130 K, the splitting is complete, and the data from a sample measured at 93 K (1-LT) fit best to a twinned structure in space group *P*1̄ (*Z* = 2). The unit cell parameters of this smaller unit cell are similar to the primitive setting of the *C*2/*c* space group of the higher-temperature model (crystallographic details in Fig. S1–S5‡).

**Fig. 3:**
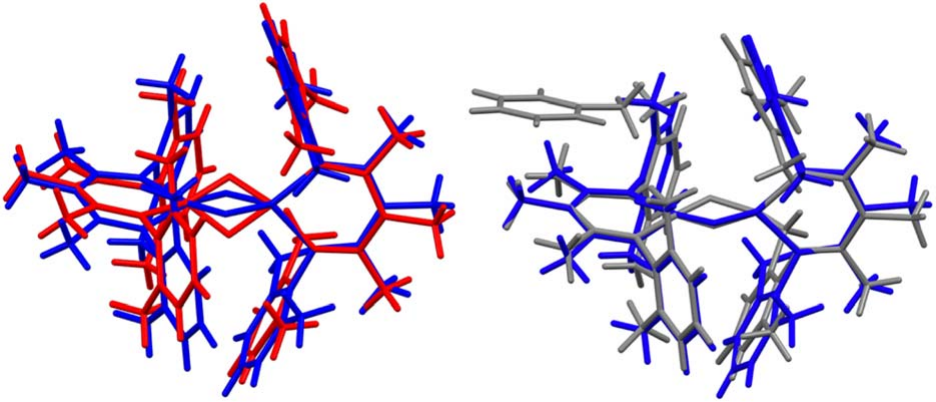
(Left) Overlay of X-ray crystal structure models of 1 at 223 K (red)^35^ and 1-LT at 93 K (blue). (Right) Overlay of X-ray crystal structure models of 1-LT at 93 K (blue) and 1-toluene at 100 K (grey). In 1, the hydrides are in (incorrect) averaged positions, whereas in 1-LT and in the structure with co-crystallized toluene (1-toluene), the hydrides are in the plane of the left Fe-diketiminate plane. Mercury files containing the overlays are available in the ESI.‡

Importantly, in 1-LT, the molecule has lost the crystallographic 2-fold axis that related the two halves of the molecule (Fig. 3). Accordingly, there is a change in the positions of the small Fourier peaks corresponding to the two bridging hydrides. However, since hydride locations are unreliable in X-ray crystallography, we sought to corroborate this structural shift using other techniques. Unfortunately, we were unable to grow a crystal of this packing form that had sufficient size for neutron crystallography.

### Decoalescence of Mössbauer spectra in the low-temperature twinned crystals

The phase change from 1 to 1-LT was accompanied by a marked change in the Mössbauer spectrum of these crystals. Cooling the crystals below 170 K results in decoalescence of the quadrupole doublet into two doublets of equal intensity (Fig. 2). The relative intensity of the two doublets is 1 : 1 at all temperatures from 150 to 5 K, and the coalescence temperature is 163(3) K. Decoalescence is characteristic of a dynamic process, and is different from spin crossover, which for mononuclear iron(ii) compounds gives the growth/disappearance of Mössbauer doublets rather than shifting and decoalescence.^41^

**Fig. 4:**
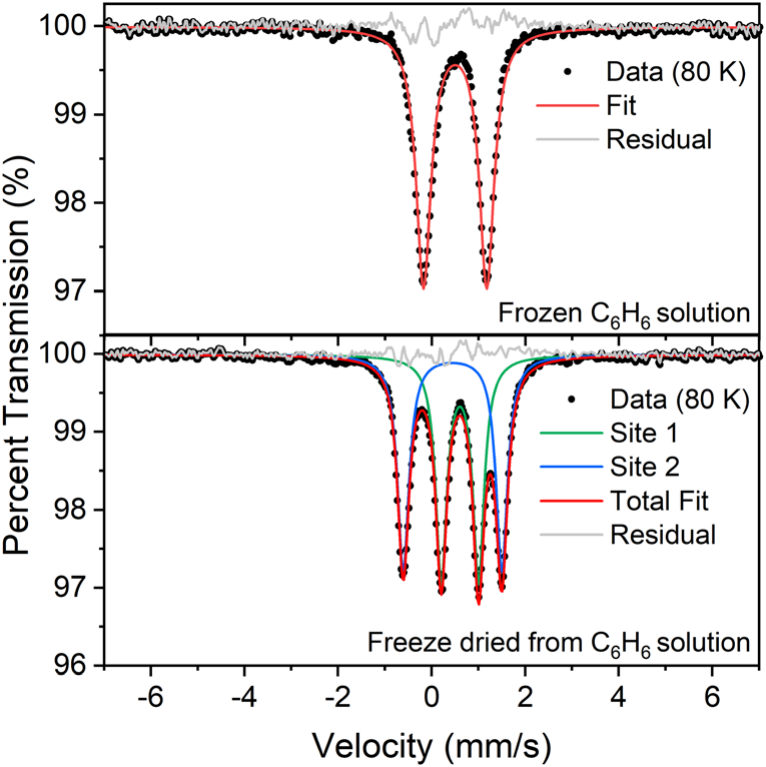
(Top) Mössbauer spectrum of a flash-frozen benzene solution of 1. Fit parameters: *δ* = 0.50 mm s^−1^, Δ*E*_Q_ = 1.35 mm s^−1^. (Bottom) Mössbauer spectrum of the same sample, after subliming away the benzene. Fit parameters: *δ* = 0.60 mm s^−1^, Δ*E*_Q_ = 0.80 mm s^−1^ (site 1, green); *δ* = 0.45 mm s^−1^, Δ*E*_Q_ = 2.11 mm s^−1^ (site 2, blue). Both spectra were recorded at 80 K.

The 1 : 1 ratio of signals at low temperature suggests that the two iron atoms in 1 are inequivalent, and the difference in isomer shifts (*δ* = 0.43 mm s^−1^*vs.* 0.60 mm s^−1^) suggests that the iron environments have different electronic structures. Further, the change in the Mössbauer spectrum at the same temperature as the change in the crystal structure indicates that this electronic structure change is linked to the crystallographic packing. We tested this idea by measuring Mössbauer spectra of flash-frozen benzene solutions. A frozen benzene solution of 1 displays only one doublet, even at 80 K. When the benzene is evaporated from this solution, the spectrum of the resulting solid at 80 K again shows two equal doublets (Fig. 4). The lack of dynamic behavior in the absence of the crystal lattice indicates that somehow the twinning of the crystals leads to the splitting of the doublets in the Mössbauer spectrum.

We further fit the dynamic behavior by considering the quadrupole splitting values, which arise from the electric field gradients (EFGs) at the two iron sites. At temperatures well below the phase transition (<100 K) they are different, and above the phase transition (>180 K) they are the same on the Mössbauer time scale (*ca.* 10^−7^ s). The coalescence behavior did not fit to a physically meaningful Boltzmann model (incoherent interconversion of sites), but they did fit to a cooperative transition (see ESI‡ for discussion). We used the Sorai–Seki domain model^42^ for a change from a static low-temperature phase to a dynamic high-temperature phase, the latter of which has fast interconversion of different possible electronic environments of the iron sites. This model has been used for entropy-driven phase transitions of mononuclear spin crossover complexes.^43^ When we adapted it for “hopping” between the environments of two iron sites, the observed temperature-dependent quadrupole splittings fit well to this model with an enthalpy factor *n*Δ*H* ∼1500 cm^−1^ (*n* represents the size of the domain; see ESI‡). In this model, the barrier for “hopping” arises from the elastic coupling of molecules in the solid, which requires a phonon coupled to the molecular motion in order to overcome the barrier. If there is no coupling between molecules (*n* = 1), the Sorai–Seki model predicts a very low barrier of ∼4 kcal mol^−1^.

### Co-crystallization with toluene and neutron crystallography

When crystals of 1 are grown from toluene rather than diethyl ether, the crystals pack differently, in a solvate form that we distinguish as 1-toluene. The Mössbauer spectrum of 1-toluene displays two doublets that have parameters similar to those observed in the low-temperature crystalline samples described above (Fig. S9‡). In the toluene crystals, the positions of the two major doublets vary only slightly with temperature, and no decoalescence is observed. X-ray diffraction studies of 1-toluene show that the overall molecular structure is like 1-LT, but with a toluene molecule stacked above one iron site. These crystals pack in the monoclinic space group *P*2_1_ with *Z* = 2, and there are no crystallographic symmetry elements within the dimeric molecules.

**Fig. 5:**
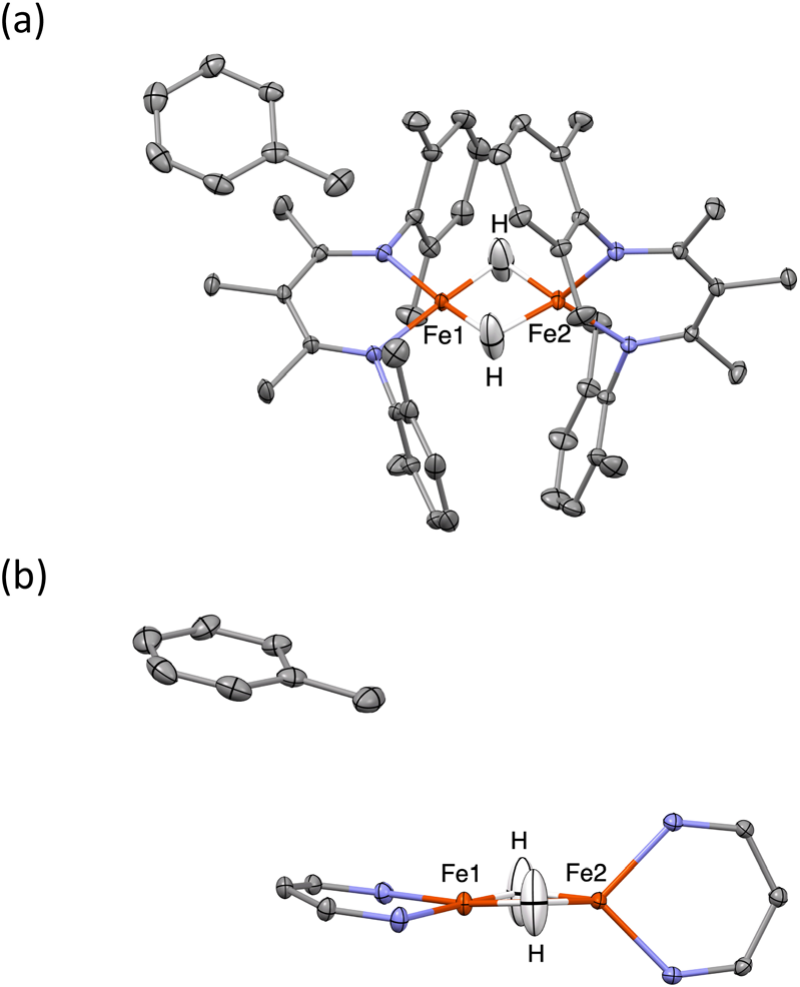
Thermal-ellipsoid plots from the neutron crystal structure of 1-toluene, using 50% probability ellipsoids. (a) View along the *C*_2_ axis. (b) View along the plane of Fe1, showing the different geometries of the sites. Diketiminate substituents are omitted for clarity.

The crystals of 1-toluene are substantially larger, enabling the use of neutron diffraction to verify the positions of the hydrogen atoms unambiguously. The neutron crystal structure of 1-toluene (Fig. 5) shows the marked difference between the coordination geometries of the two inequivalent iron atoms. Fe1 has a square-planar geometry (5.6(1)° between the NFeN and HFeH planes), and the Fe1–H distances of 1.62(1) and 1.64(1) Å are significantly shorter than the Fe2–H distances to pseudotetrahedral (89.7(1)° between planes) Fe2 at 1.71(1) and 1.73(1) Å. The parameter *τ*_4_, which quantifies the geometry of a metal center on a scale from 0 (square planar) to 1 (tetrahedral) is 0.06 for Fe1 and is 0.80 for Fe2. The Fe–N distances at Fe1 are also shorter (1.924(4) and 1.925(3) Å) than to Fe2 (1.936(3) and 1.967(3) Å), suggesting that Fe1 has a lower spin state than Fe2.

**Fig. 6:**
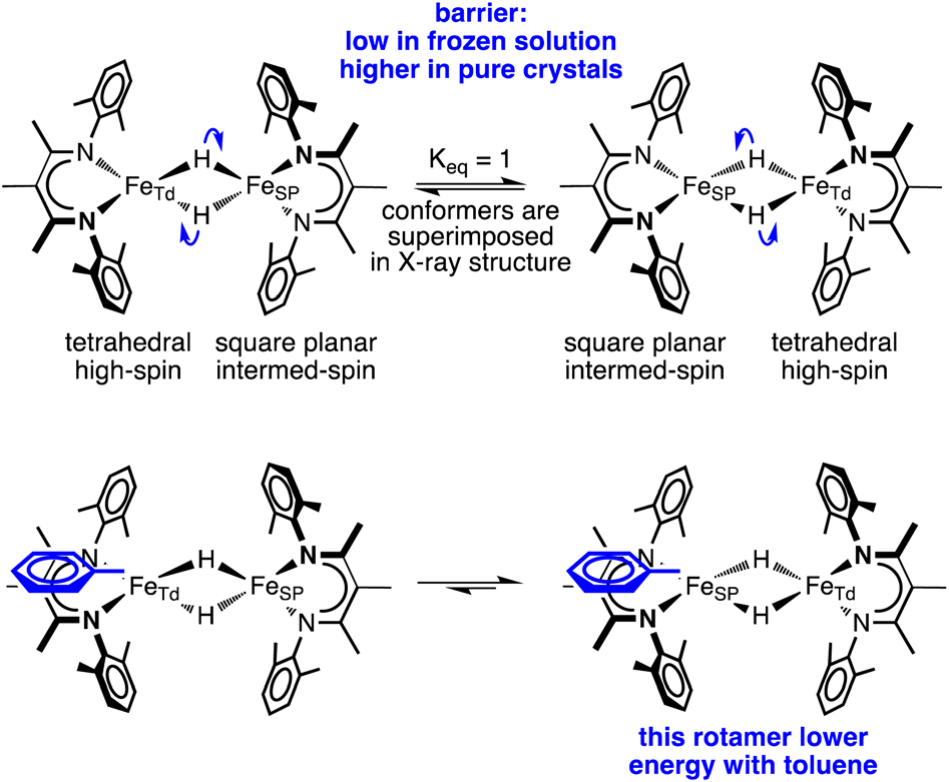
A model of hydride motions in 1 that explains the accumulated spectroscopic, crystallographic, and magnetic data.

The crystal structures of 1-toluene show that the toluene of crystallization is stacked above the β-diketiminate of the square-planar Fe1, with its methyl group wedged between the xylyl groups of the supporting ligand (methyl carbon 3.7 Å from plane, and H 2.7 Å from plane). This toluene molecule in the crystal does not change the electronic structure, as shown by the similarity of the Mössbauer parameters between crystals of 1-LT and 1-toluene (Fig. S9‡). Instead, the toluene molecule in the crystal influences the relative energies of the two geometries, as shown in the bottom of Fig. 6. In this model, the presence of a flat toluene molecule near only one of the two iron sites “freezes out” one of the two conformational possibilities. This differs from toluene-free crystals of 1, where the two conformations (Fe1_SP_/Fe2_Td_ and Fe1_Td_/Fe2_SP_) can interconvert with very little motion of the supporting ligands (Fig. 3).

This logic leads to the following overall explanation for both the Mössbauer and crystallographic observations in 1 (Fig. 6). At higher temperatures in the higher-symmetry space group *C*2/*c*, the iron and hydride positions are crystallographically required to be equivalent due to the 2-fold rotation axis, but the “observed” positions are an artifact arising from multiple geometries of the molecular cores in these crystals. Near 150 K, there is a phase change to a lower-symmetry space group, which removes the crystallographic 2-fold axis. Half of the domains rotate the hydrides in each direction, which leads to twinning in 1-LT (Fig. S4 and S5‡). The observation of 50 : 50 twinning suggests that this desymmetrization follows the same rotation within domains, rather than alternating molecules in the crystal. This domain-wide motion requires coherent reorganizations (phonons), which enable the molecules to overcome the barrier.

In 1-toluene, the two Fe sites are crystallographically inequivalent because of the packing of the toluene molecule above one of the two iron-diketiminate units. There is no symmetry breaking of the molecule with temperature in crystals of 1-toluene, since the iron sites are already distinct at all temperatures in the crystal due to the location of the toluene. This explains why 1-toluene crystals show two quadrupole doublets in Mössbauer spectra at all temperatures.

In contrast, in the flash-frozen benzene solution the molecules have disorganized molecular surroundings. Because there is no cooperativity in the interconversion of the conformers in the crystal, the barrier is low for fast interchange of the local electronic environment between Td and SP geometries (*τ* ≪ 10^−8^ s) and therefore averaged Mössbauer spectra are seen at all temperatures. This model is consistent with DFT computations below, which show that the barrier to rotation of the Fe_2_H_2_ plane within the ligand scaffold is only 3 kcal mol^−1^ in the absence of intermolecular interactions.

Finally, to evaluate the dependence of the dynamics on the isotope of hydrogen, we compared the temperature dependence of the Mössbauer spectrum for crystals of 1 and its deuteriated analogue 1-D (Fig. S12 and S13‡). The observed coalescence temperatures were the same for the hydride and deuteride within 3 K. The lack of an observable kinetic isotope effect supports the idea that the phase change is the driver of the temperature-dependent changes, whereas motion of the hydrides is rapid in the absence of the twinned 1-LT crystal packing. We also collected a neutron structure of the toluene crystals from 1-D, and the metrical parameters match the protiated structure within standard uncertainty limits, despite the potential for differences between the size of H and D.^44^

### Spin states of the iron sites

**Fig. 7:**
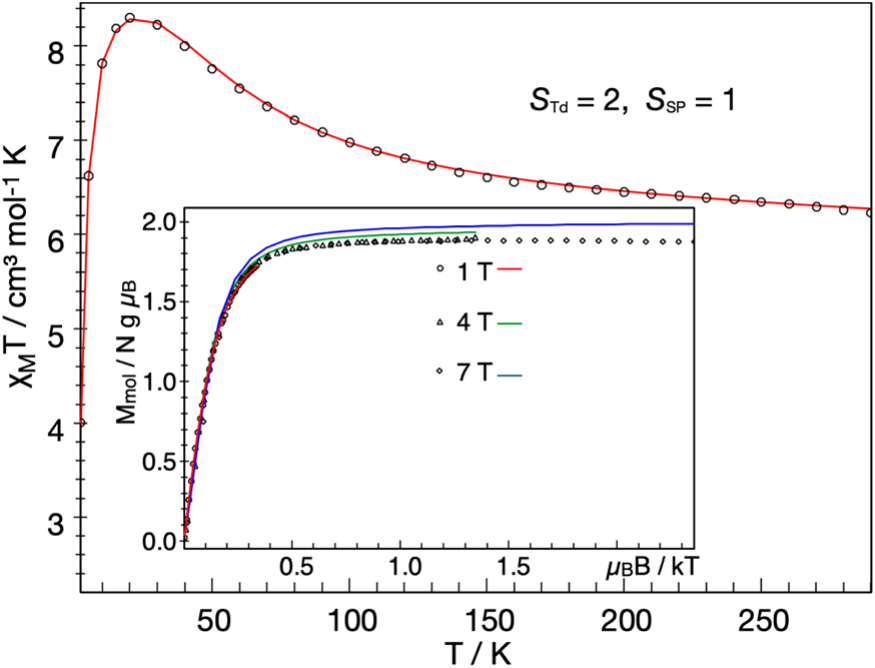
Temperature dependence of the molar magnetic susceptibility times temperature, *χ*_M_*T*, of 1 recorded under an applied field of 1 T, and inverse temperature dependence of the magnetization recorded with 1, 4 and 7 T (inset). The experimental data were corrected for a TIP-like contribution to *χ* of 600 × 10^−6^ cm^3^ mol^−1^. The colored lines represent a global SH simulation using *S*_Td_ = 2, *S*_SP_ = 1, *J* = +63 cm^−1^, *g*_Td_ = (2.47, 2.47, 2.61), *g*_SP_ = (3.65, 3.65, 1.0), *D*_Td_ = −26.1 cm^−1^, and *D*_SP_ = +40 cm^−1^. The rhombicity parameters were constrained to 
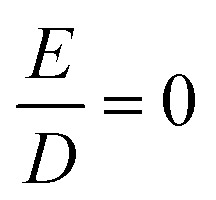
 and the ***D*** and ***g*** matrices of the Fe_SP_ site are rotated with respect to the principal axes for the Fe_Td_ site by an Euler angle *β* = 98° in this model. See ESI‡ for details of the model.

The solid-state magnetic moment of a powder freeze-dried from benzene was measured as a function of temperature, and the magnetization data are shown in Fig. 7. There is no discontinuity at the temperature of the crystallographic phase change (163 K). There is a peak in the plot of *χ*_M_*T versus T*, and the high-temperature asymptote is near 6.2 cm^3^ mol^−1^ K (near the spin-only value of 6.0 expected for *S* = 3). Isofield magnetization measurements at 1, 4 and 7 T and recorded on an inverse temperature axis show almost no nesting (inset of Fig. 7). This reveals the ground state to be a small set of energetically well isolated magnetic sublevels with a large negative zero-field splitting (zfs) that renders the highest *m*_s_ levels lowest in energy.

**Fig. 8:**
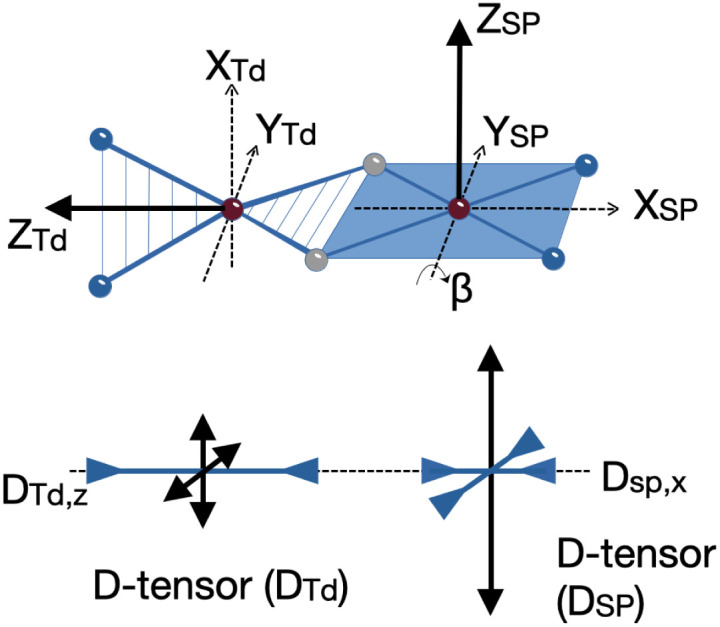
(Top) Schematic view of the rotated magnetic axes for the square planar (SP) relative to the tetrahedral (Td) sites of 1 as obtained from the simulation of the magnetic data. (Bottom) Relative orientation of the ***D***-tensors for Td and SP sites. The double arrows pointing outward and inward indicate the positive and negative tensor components, respectively. Note that the ***D***-tensors are traceless in the SH formalism, *i.e.* the total length of positive and negative double arrows is zero. Analogous plots would hold for ***g***-matrices, electric field gradient tensors (*V*_ij_), as well as magnetic hyperfine coupling tensors ***A*** for ^57^Fe.

We could fit these data to three models: (a) two *S* = 2 sites with ferromagnetic coupling and a diamagnetic impurity, (b) one *S* = 2 subsite and one *S* = 1 subsite, or (c) one delocalized *S* = 3 system. The first model did not fit our magnetic Mössbauer data; see ESI‡ for details and description of the alternative model. The second model yielded *S*_Td_ = 2, *g*_Td_ = 2.47, 2.47, 2.61, and *D*_Td_ = −26 cm^−1^, and *S*_SP_ = 1, *g*_SP_ = 3.65, 3.65, 1.0, and *D*_SP_ = +40 cm^−1^, with exchange coupling constant *J* = +63 cm^−1^ (using −2*J S*_Td_·*S*_SP_). The model required different orientations of the two local ***D*** and ***g*** matrices, as shown in Fig. 8. Spin projection arguments show that the zero-field splitting of the tetrahedral site *D*_Td_ is the main source of magnetic anisotropy (see ESI‡ for details).

Aligning the ***g***_Td_ and ***g***_SP_ matrices and the local ***D***-tensors as shown in Fig. 8 reproduced the experimental over-shooting of *χ*_M_*T*(*T*) around 20 K (red fit in Fig. 7). The large *g* components along *z*_Td_ strengthen the magnetization of the (*z*-polarizable) high *m*_s_ levels, which are exclusively populated at low temperatures. In contrast, lower *g*_Td_ values in the *x*/*y* direction reduce the magnetization of the other levels with lower *m*_s_ that become considerably populated above 20 K. This difference in magnetization of the *m*_s_ levels causes the observed overshooting of *χ*_M_*T*(*T*) with temperature, rather than the more typical assignment of overshooting to ferromagnetic coupling.

We also modeled the magnetization in the context of an isolated *S*_total_ = 3 system. This model also provides a reasonable global fit of magnetic susceptibility and magnetic Mössbauer data (Fig. S17‡). The zfs parameter of the *S* = 3 model, *D* = −11 cm^−1^, is consistent with *D*_Td_ = −26 cm^−1^ in the localized model (due to the projection coefficient 2/5). In the delocalized model, the over-shooting of *χ*_M_*T*(*T*) around 20 K is caused entirely by strong *g* anisotropy, *g* = (1.4, 1.4, 2.6). The same behavior was previously observed for an *S* = 1 {FeNO}^8^ species^45^ and a two-coordinate *S* = 2 imidoiron(ii) species,^46^ both of which feature large negative *D* and *g*_*z*_ larger than 2. Large and anisotropic shifts in *g* values have been observed previously in mononuclear *S* = 1 square-planar iron(ii) compounds, such as [Fe^II^(TPP)].^47^ Further details of the various models may be found in the ESI.‡

### Electronic structure from computations

To gain further insight into the electronic structure of 1, we undertook quantum chemical studies. The geometry-optimized computational models at the BP86/def2-TZVP, B3LYP/def2-TZVP and TPSSh/def2-TZVP levels of theory unanimously demonstrated the *S* = 3 state to be lower in energy than the *S* = 4 state that would come from two ferromagnetically coupled high-spin iron(ii) sites.^48^ The *S* = 3 model at the neutron crystallographic geometry predicted Mössbauer parameters accurately (*δ*_calc_ = 0.57 (Td), 0.45 (SP) *vs. δ*_exp_ = 0.60, 0.43; Δ*E*_Qcalc_ = 1.29 (Td), 1.55 (SP) *vs.* Δ*E*_Qexp_ = 0.74, 2.16; Table S4‡), and was used for the more detailed information on the electronic structure.

**Fig. 9:**
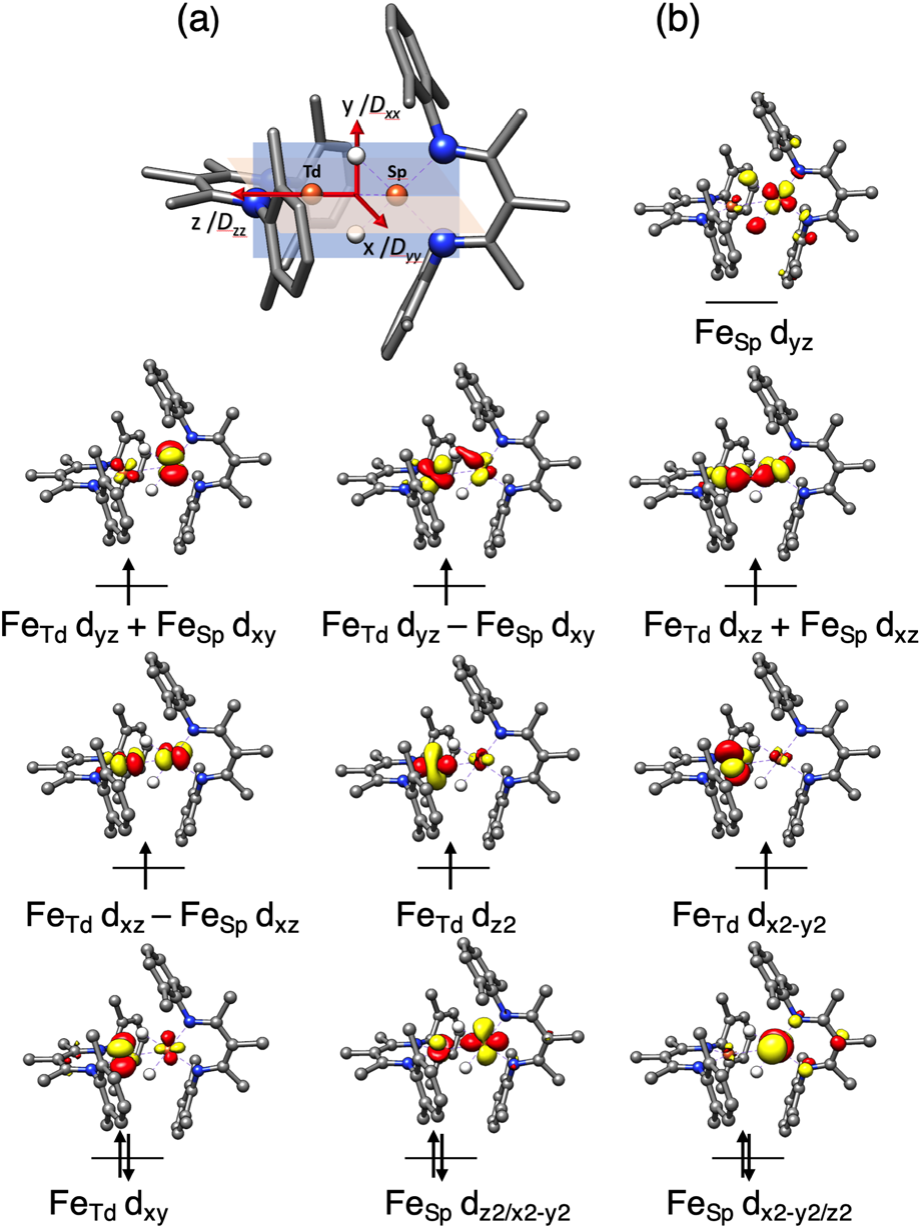
(a) Orientation of the ***D*** tensor, and (b) active orbitals of 1, obtained from CASSCF(14,11) calculations averaging three low-lying *S* = 3 states. The orbital labels follow the localized axes of Fig. 8.

To elucidate the physical origin of the strong magnetic anisotropy of 1, it is necessary to explicitly consider spin–orbit coupling (SOC) of its low-lying electronic states. To this end, we carried out wavefunction-based CASSCF/NEVPT2 calculations (Fig. 9). The reference frame was chosen with the *z* axis along the Fe–Fe vector, and the *xz* and *yz* planes as the N_Td_Fe_Td_N_Td_ and N_SP_Fe_SP_N_SP_ planes, respectively. The active space spanned both Fe sites, and included all Fe 3d orbitals of the Fe_Td_ and Fe_SP_ sites as well as the σ-bonding counterpart of the high-lying Fe_SP_ d_*yz*_ based orbital (not shown in Fig. 9b). These CASSCF(14,11) calculations predicted that 1 has three *S* = 3 states within 700 cm^−1^, which indicates that the system possesses an orbitally near-degenerate ground state. (As a consequence, we did not succeed in converging single-root ground-state CASSCF calculations as CASSCF computations suffered from severe convergence problems, but had to average the three low-lying septet states.) The septet ground state of 1 has substantial multireference character, because each of the electron configurations accounts for less than 16% of the wavefunction.

The CASSCF(14,11)/NEVPT2 computations give *D* = −12.9 cm^−1^ and *E*/*D* = 0.29, which is in reasonable agreement with experiment (*D*_total_ = −11 cm^−1^). The largest component (*D*_*zz*_) is along the *z* axis (Fe–Fe vector), and the smallest *D*_*xx*_ and medium *D*_*yy*_ components as defined in Fig. 9a. The principal axis systems of both ***D*** and ***g*** matrices are calculated to be almost collinear and *g*_*x*,*y*,*z*_ = 1.97, 2.29, 2.60 with respect to the reference frame. The three lowest-energy excited states (Table S5‡) make the dominant contributions to *D*. The first excited state lies 390 cm^−1^ higher in energy and largely stems from the single excitation Fe_Td_ d_*xy*_ → d_*x*^2^–*y*^2^_. Hence, the Fe_Td_ site is the main contributor to an orbitally nearly degenerate ground state. The in-state SOC introduces a negative *D* value at the Fe_Td_ site, and therefore leads to its easy-axis magnetic anisotropy along the *z* axis in agreement with the fitting of the magnetic data presented above. A closely related mononuclear pseudotetrahedral high spin ferrous complex, LFeCl_2_Li(THF)_2_ (L = β-diketiminate), also features an analogous electronic structure and hence similar magnetic properties.^49^ The second and third excited states were computed to lie 770 and 1250 cm^−1^ above the ground state, respectively, and predominantly arise from single excitations of Fe_SP_ d_*x*^2^–*y*^2^_ → d_*xz*_ and d_*x*^2^–*y*^2^_ → d_*xy*_. The SOC of these excited states with the ground state leads to considerable orbital angular momentum in the *yz* plane, in agreement with the rotation of the Fe_SP_***D*** tensor presented above. Furthermore, because the aforementioned excitations are all DOMO-to-SOMO transitions (DOMO = doubly occupied molecular orbital, SOMO = singly occupied molecular orbital), the resulting *g*-shifts are positive and agree with the experimentally observed *g*_*y*_ and *g*_*z*_ values that are considerably greater than 2. The fact that the three dominant excitations are each localized to one of the two sites adds weight to a localized model of the electronic structure, as discussed below.

### Dynamics from computations

**Fig. 10:**
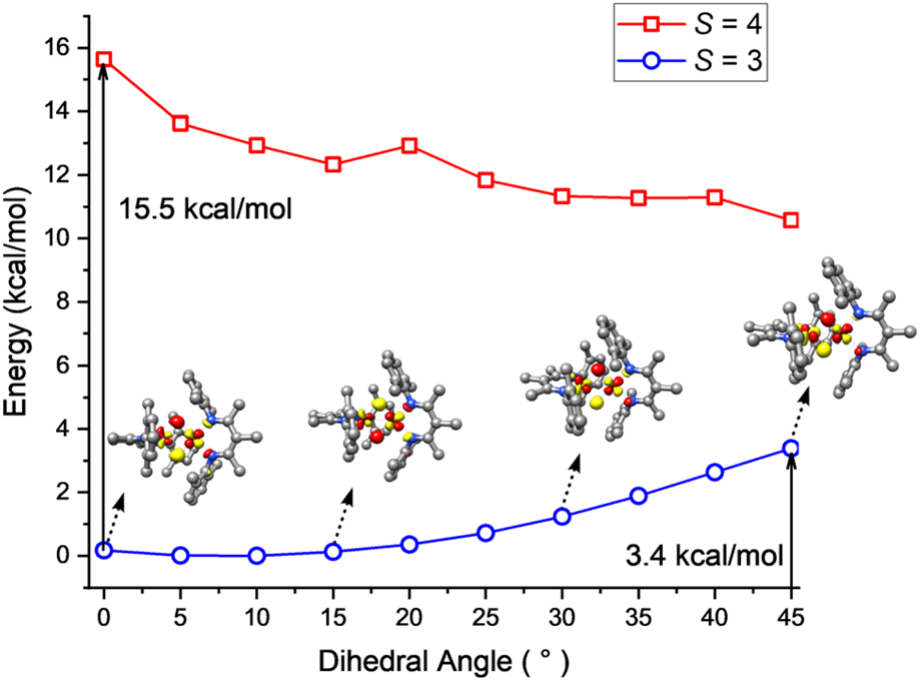
B3LYP-computed energy variations of the septet (blue) and nonet (red) states as a function of the dihedral angle between the N_SP_Fe_SP_N_SP_ and HFe_SP_H planes along with the lowest-energy unoccupied molecular orbitals (LUMO) of the *S* = 3 state at selected dihedral angles.

To shed light on the dynamic behavior, we used DFT (B3LYP/def2-TZVP level) to calculate a relaxed potential surface in which the dihedral angle *θ* between the N_SP_Fe_SP_N_SP_ and Fe_SP_HFe_Td_H planes was systematically varied (Fig. 10). The *S* = 3 model reproduces the experimental geometry, while the *S* = 4 model prefers a dihedral angle *θ* of 45°. However, the *S* = 4 model never goes low enough in energy to become the ground state, in agreement with the magnetization fits described above. For the *S* = 3 model, B3LYP computations showed a low barrier of 3–4 kcal mol^−1^ (at the transition state with *θ* = 45°) for interconversion of the geometries of the two sites. The lack of a high reorganization barrier suggests that the hydride dimer can rotate rapidly in the absence of intermolecular interactions, consistent with the averaging of Mössbauer quadrupole doublets of the Fe_SP_ and Fe_Td_ sites in frozen solutions. This supports the idea advanced above, that the phase change is the rate-limiting step for the reversal of Fe_SP_ and Fe_Td_ sites because it has a higher barrier than hydride motions. This in turn is consistent with the lack of an H/D kinetic isotope effect in the Mössbauer coalescence.

The DFT calculations also give information about how the electronic structure responds to geometric changes. At a dihedral angle *θ* near zero, the lowest-energy unoccupied molecular orbital (LUMO) of 1 is mostly derived from a Fe_SP_ d_*yz*_ orbital with limited contribution from the Fe_Td_ d_*yz*_ orbital. With increasing *θ*, the Fe_Td_ d_*yz*_ parentage gradually increases at the cost of Fe_SP_ d_*yz*_. This change culminates when *θ* = 45°, at which both fragment orbitals make approximately equal contributions to the LUMO. Because of this smooth transition, the exchange of the local electronic configurations between the two Fe centers does not require electronic excitation, and proceeds with a low barrier.

## Discussion

### Perspectives

This is a unique system in which there is rapid, coupled interconversion of geometries and local electronic configurations between two nearby iron sites. These can trade local tetrahedral (Td, favors high-spin) and square planar (SP, favors intermediate-spin) geometries. Since the supporting ligands on the two metals are perpendicular, making one site square planar forces the other to be tetrahedral and *vice versa*. The movement of the bridging hydrides changes the geometry of both sides, which could explain why the overall spin state does not change: when one side changes from tetrahedral (local high spin *S*_Td_ = 2) to square planar (local intermediate spin *S*_SP_ = 1), the other side makes the opposite change simultaneously, leaving *S*_total_ = 3. Computational studies show that motion of the hydrides entails a smooth exchange of local electronic structures that contributes to this low barrier.

However, this picture assumes a spin Hamiltonian model with ferromagnetic coupling between two discrete spins (model b from "Spin states of the iron sites" above). This model fit all of the available data with *J* = +63 cm^−1^. However, given the strong electronic coupling between the sites, it is also possible to view the septet ground state as a single *S* = 3 spin system that is delocalized across both metal sites (model c above). This alternative model did not fit the magnetic susceptibility data quite as well, as it corresponds to an unmeasurably large value of *J*, but there could also be limitations due to the zfs model we used. Though the CASSCF natural orbitals above appear delocalized, the in-plane Fe_SP_ d_*yz*_ orbital is unpopulated in the ten lowest-energy *S* = 3 states within 3000 cm^−1^, as expected from qualitative ligand field theory for a square-planar Fe_SP_ site. As noted above, the lowest-lying excitations were localized on the tetrahedral Fe_Td_ site. Thus, aspects of the electronic structure are localized. Overall, this system is at the borderline of systems that are best treated with a delocalized model, which explains spin alignment through Hund's rule as in the multi-iron complexes of Betley.^50^ Here, mixing is facilitated by the short distance between the metals (2.45 Å, close to Pauling's Fe–Fe single bond distance of 2.48 Å).^51^ Berry has described a dinickel complex in which two bridging hydrides similarly enforce close metal–metal contacts that lead to a non-intuitive ground spin state.^52^

The geometry, electronic structure and magnetism of 1 bear a close similarity to a bis(carbene)-supported diiron system recently reported by Smith and coworkers (2), which has two hydride bridges.^53^ Though neutron crystallography was not used, an X-ray crystal structure suggested one square-planar iron(ii) site and one tetrahedral iron(ii) site as shown here, and the Mössbauer spectra strongly supported this interpretation. Like 1, their compound had an *S* = 3 ground state that was described through a ferromagnetic coupling model, but the fitted exchange constant was *J* = +110 cm^−1^. This corresponds even more closely to a fully delocalized septet. They also used high-field EPR spectroscopy to derive an overall *D* = −7 cm^−1^ which is somewhat less than in 1 (−11 cm^−1^), presumably because the stronger-field carbene ligands more effectively quench the orbital angular momentum. However, the frontier orbitals (derived from DFT in their work) bear great resemblance to the ones derived here. In the bis(carbene) supported system, the structure and Mössbauer doublets showed distinct iron sites at all investigated temperatures, and thus were not subject to the phase change dependence described here.

The most unusual aspect of our findings is the topotactic transition^54,55^ around 160 K, in which the low-symmetry twinned unit cell at low temperature changes to a higher-symmetry untwinned unit cell with crystallographic equivalence between the two iron sites. At low temperature, the crystallographic differentiation of the iron sites gives an “extra” barrier for hydride motion, because trading the geometries of the two sites cannot be done one molecule at a time. Rather, movement of the hydrides to trade site geometries requires a coordinated motion of an entire domain! This may seem surprising since the hydrides are small and the supporting ligands do not change orientations, but there are substantial differences between the Fe–N(diketiminate) bond lengths of the tetrahedral and square planar sites, which are apparently sufficient to influence the intermolecular interactions. Then, the relaxation of crystal packing constraints above 160 K removes the intermolecular forces, giving a low barrier for hydride movement and coalescence on the Mössbauer timescale.§§The shapes of the Mössbauer signals were the same when approaching a given temperature from the warmer or colder direction. Thus the phase change in 1 is fully reversible. Both DFT and extrapolation of the Sorai–Seki model suggested a very low barrier of <4 kcal mol^−1^ for hydride rotation around the Fe–Fe axis in the absence of crystal packing forces. Quantum tunneling would be expected to affect the barrier, but we were unable to assess it experimentally because the rates measured here arose solely from the phase change; without this constraint, the hydride motion was too rapid to measure using methods at our disposal.

### Implications

These results illustrate the strong influence on electronic structure and spin state that can arise from the dynamic nature of hydrides. First, small bridging hydrides may hold two iron centers extremely close together, which leads to orbital mixing and communication between spins on the two sites.^52,53^ Second, hydrides can move with little energy cost, as a result of bonding through nondirectional 1s orbitals.^56,57^ In the system studied here, the supporting ligands do not need to move, and the hydrides can rotate around the Fe–Fe vector in a way that maintains a rhomboidal Fe_2_H_2_ core while accessing the full range of dihedral angles. Third and most crucially, hydride ligands are strong-field, so small movements can give large changes in d-orbital energies (manifested here as a change in local spin state).

It is interesting to speculate about a connection to recent work on the FeMoco of nitrogenase, in which reduced forms E_2_ and E_4_ have been shown to have bridging hydride ligands and have accordingly been termed E_2_(2H) and E_4_(4H).^11–14^ In particular, the E_4_(4H) state responds to N_2_ binding by undergoing reductive elimination of H_2_ from these hydrides, which provides an essential driving force to move along the pathway toward ammonia formation.^13^ However, research on nitrogenase rarely considers the ligand field that is induced by these hydrides.^14,58–61^ We suggest that hydrides on the FeMoco might offer a powerful combination of (a) extreme mobility^56^ and (b) ability to strongly modulate the relative energies of iron orbitals (perhaps even changing the iron sites from the high-spin configuration of the resting state to a lower-spin configuration).^59^ In both nitrogenase and in future catalytic systems, we suggest that hydrides have the potential to be used as supporting ligands for rapid access to diverse electronic structures and spin, and thus their role may transcend the reactions that hydrides themselves perform.

## Conclusions

In this diiron(ii) system, strong interactions between very close hydride-bridged iron sites lead to unusually large ferromagnetic coupling in a model that uses localized spin sites. Rapid motions of the bridging hydride ligands, though, cause changes in the local electronic structure at these two iron sites. Due to variable crystal packing, it is possible to freeze out the motion and to gain unprecedented detail into the spin flipping phenomena. The major changes in ligand-field and magnetic properties from this rapid movement of hydrides suggests that incorporation of bridging hydrides could be a generally useful method for modulating the electronic structures of metal sites. We attribute this to the uniquely small size, high mobility, and strong ligand field of bridging hydrides. For example, nitrogenases may use bridging hydrides in the FeMoco not only for the previously recognized purposes like reductive elimination of H_2_, but also to bring about rapid ligand-field changes that change spin states of iron sites or facilitate reactions of substrates.

## Data availability

Crystallographic data for the compounds have been deposited at the CCDC, and the deposition codes are in the ESI. Other data for this paper are also in the ESI.†

## Author contributions

K. C. M. and S. F. M. performed the synthesis, NMR, and Mössbauer experiments. S. F. M., B. Q. M., and X. W. performed crystallographic measurements. M. S. F. and E. B. performed and interpreted magnetic measurements. M. T. and S. Y. performed calculations. E. B. and S. Y. modeled the electronic structure including the dynamical features. P. L. H. wrote the paper and supervised the project.

## Conflicts of interest

There are no conflicts to declare.

## Supplementary Material

SC-014-D2SC06412J-s001

SC-014-D2SC06412J-s002
