# Supplementary material for: Dynamic effects on ligand field from rapid hydride motion in an iron(ii) dimer with an S = 3 ground state
Source: Chem Sci. 2023 Feb 8;14(9):2303–12. doi: 10.1039/d2sc06412j (PMC9977447; doi:10.1039/d2sc06412j)
Supplement: SC-014-D2SC06412J-s001 [file SC-014-D2SC06412J-s001.pdf]

Electronic Supplementary Material (ESI) for Chemical Science.
This journal is © The Royal Society of Chemistry 2023

Supporting Information

Dynamic Effects on Ligand Field from Rapid Hydride Motion in an Iron(II) Dimer with an S=3 Ground State

Sean F. McWilliams, Brandon Q. Mercado, K. Cory MacLeod, Majed S. Fataftah, Maxime Tarrago, Xiaoping Wang, Eckhard Bill, Shengfa Ye*, Patrick L. Holland**

Preparation of 1 followed the method given in the literature.[1] Samples of **1-D** were prepared by placing a sample of **1** under 1 atm of D_2 three times, as previously described.[2] All manipulations of solids and solutions were performed with careful exclusion of air and moisture, using M. Braun gloveboxes maintained at < 1 ppm of O_2. Solvents were dried by passage through alumina columns (Glass Contour Co.) and stored on activated molecular sieves under an atmosphere of N_2. ^{1}H NMR spectra were recorded on Agilent NMR spectrometers operating at 400.13 MHz.

Zero-field Mössbauer spectra were recorded on a SEE Co. MS4 Mössbauer spectrometer in the constant acceleration mode with a 0.07 T applied magnetic field. The spectrometer is integrated with a Janis SVT-400T He/N_2 cryostat. Isomer shifts were determined relative to a metallic foil of α–Fe collected at room temperature. The zero-field spectra were simulated using Lorentzian doublets using WMoss (SeeCo).

Table S1: Details of Crystal Structures **1-LT**, **2-toluene**, **1-H-neut**, and **1-D-neut**

**Compound**	**1-LT**	**1-toluene**	**1-H-neut**	**1-D-neut**
**Data Code**	007-14051	als-15015	FeH	FeD
**CCDC Number**	1940222	1940223	1940224	1940225
**Empirical Formula**	$C_{44}H_{56}Fe_2N_4$	$C_{102}H_{128}Fe_4N_8$	$C_{51}H_{64}Fe_2N_4$	$C_{51}H_{62.17}D_{1.83}Fe_2N_4$
**Temperature (K)**	93(2)	100(2)	100(2)	100(2)
**Wavelength (Å)**	1.54184	0.7749	0.4-3.5	0.4 – 3.5
**Crystal System**	Triclinic	Monoclinic	Monoclinic	Monoclinic
**Space Group**	$P\bar{1}$	$P2_1$	$P2_1$	$P2_1$
a **(Å)**	11.1005(4)	11.9085(4)	11.8026(3)	11.8009(5)
b **(Å)**	11.3249(4)	11.0443(4)	10.9628(3)	10.9639(4)
c **(Å)**	18.9146(4)	17.4990(7)	17.4178(5)	17.4087(7)
**α (º)**	103.931(2)	90	90	90
**β (º)**	93.575(2)	94.175(2)	94.360(3)	94.329(4)
**γ (º)**	116.835(4)	90	90	90
V **($Å^3$)**	2018.76(13)	2295.38(15)	2247.16(11)	2245.98(16)
Z	2	2	2	2
ρ **(g/cm^3)**	1.238	1.222	1.248	1.252
μ **(mm^{-1})**	6.012	0.846	(0.1490 + 0.1103λ)	(0.1465 + 0.1068λ)
**Completeness (%)**	99.6	99.9	96.6	94.1
**Resolution (Å)**	0.83	0.83	0.78	0.78
**Data / restraints / parameters**	15047 / 0 / 474	8334 / 1 / 537	34387 / 1 / 1113	23195 / 212 / 1098
***R*1, w*R*2 (I > 2*σ*(I))**	0.0708, 0.2625	0.0291, 0.0658	0.0568, 0.1134	0.0641, 0.1191
***R*1, *wR*2 (all data)**	0.0909, 0.2948	0.0338, 0.0679	0.0633, 0.1175	0.0689, 0.1222
**GOF**	1.031	1.041	1.025	1.050
**Largest Diff. Peak, Hole**	1.004, -0.615 (e $Å^{-3}$)	0.147, -0.195 (e $Å^{-3}$)	1.187, -0.814 (fm $Å^{-3}$)	1.067, -1.241 (fm $Å^{-3}$)

Experimental, refinement and model details for 1-LT. Low-temperature diffraction data (ω-scans) were collected on a Rigaku MicroMax-007HF diffractometer coupled to a Saturn994+ CCD detector with Cu Kα (λ = 1.54178 Å). The diffraction images were processed and scaled using the CrysAlisPro software package (Rigaku OD, 2015). The structure was solved with SHELXT and was refined against F^2 on all data by full-matrix least squares with SHELXL (Sheldrick, G. M. *Acta Cryst.* **2008**, *A64*, 112–122). These data were refined as a 2-component twin with the twin law 0 1 0 1 0 0 -1 -1 -1. The two domains are related by a 180° rotation about the center of reciprocal space. The fractional volume contribution of the minor twin component was freely refined to a converged value of 0.465(2). All non-hydrogen atoms were refined using anisotropic thermal parameters. The isotropic displacement parameters of all hydrogen atoms were fixed to 1.2 times the *U* value of the atoms to which they are linked (1.5 times for methyl groups). Most hydrogen atoms were included in the model at calculated positions and refined using the riding model described above. The only exceptions are H1 and H2, which were found in the difference map and freely refined. The full numbering scheme of compound **1-LT** can be found in the CIF. CCDC number 1940222 contains the supplementary crystallographic data for this compound.

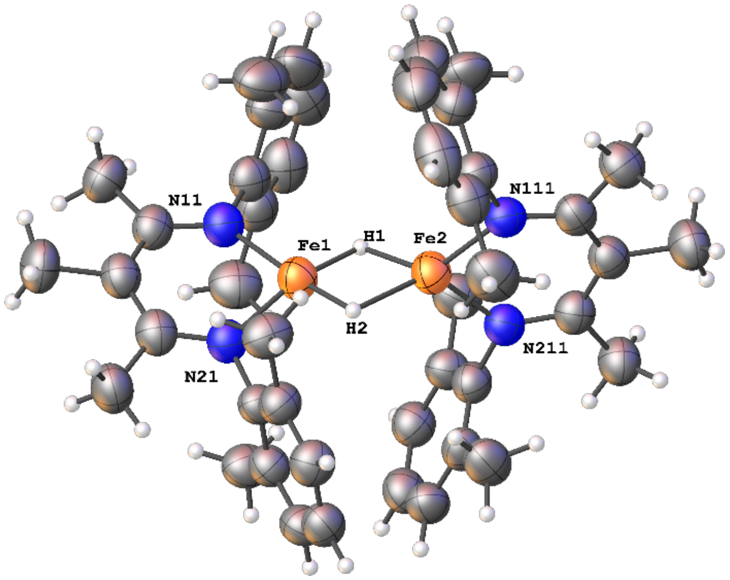

Figure S1. A partial numbering scheme of **1-LT** with 50% probability thermal ellipsoids. The hydrogen atoms are shown as spheres with arbitrary size.

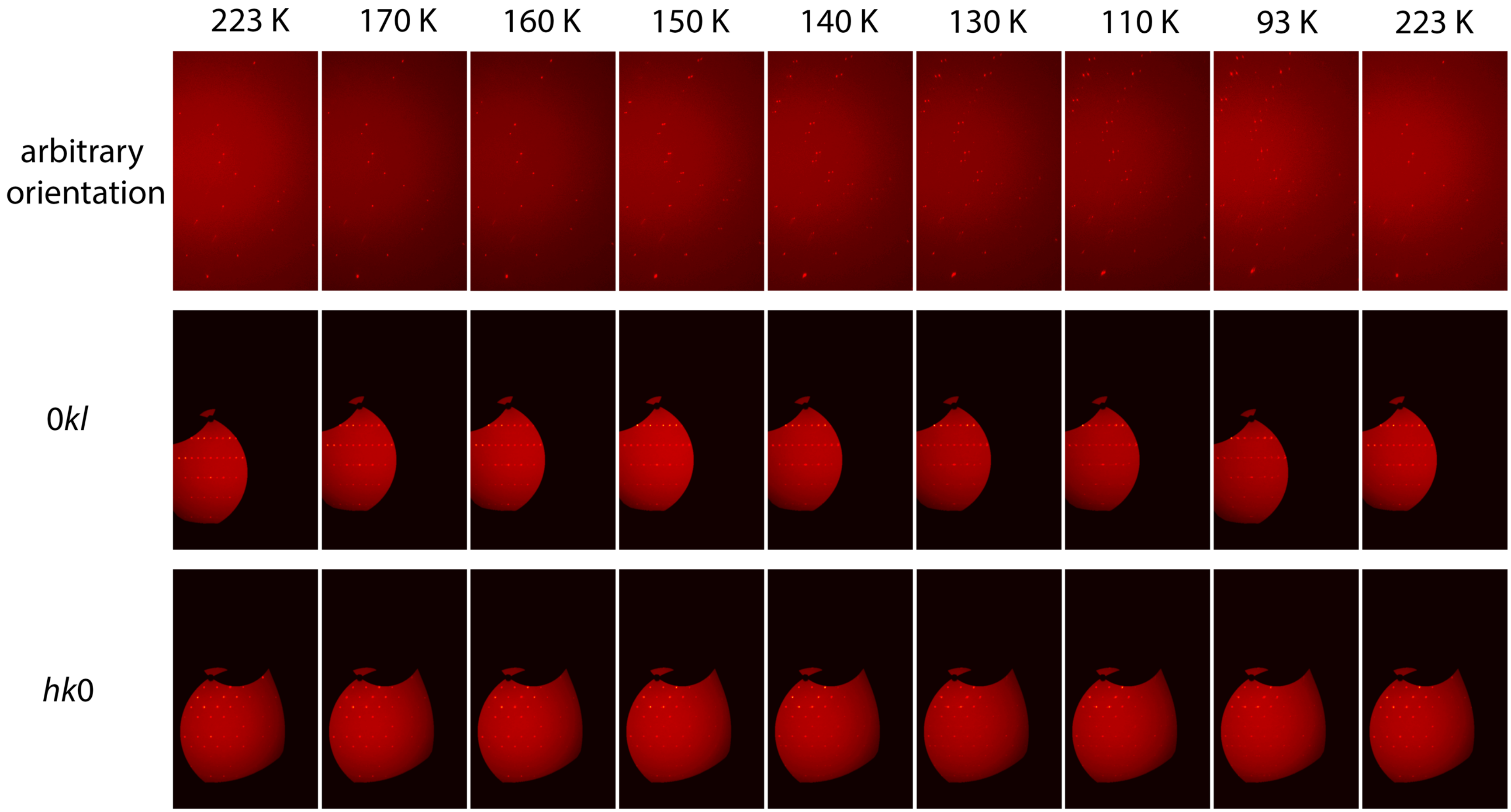

Figure S2. Rotation photographs as a function of temperature during the phase change of **1** to **1-LT**. Note that the split diffraction spots return to the original unsplit spots upon warming.

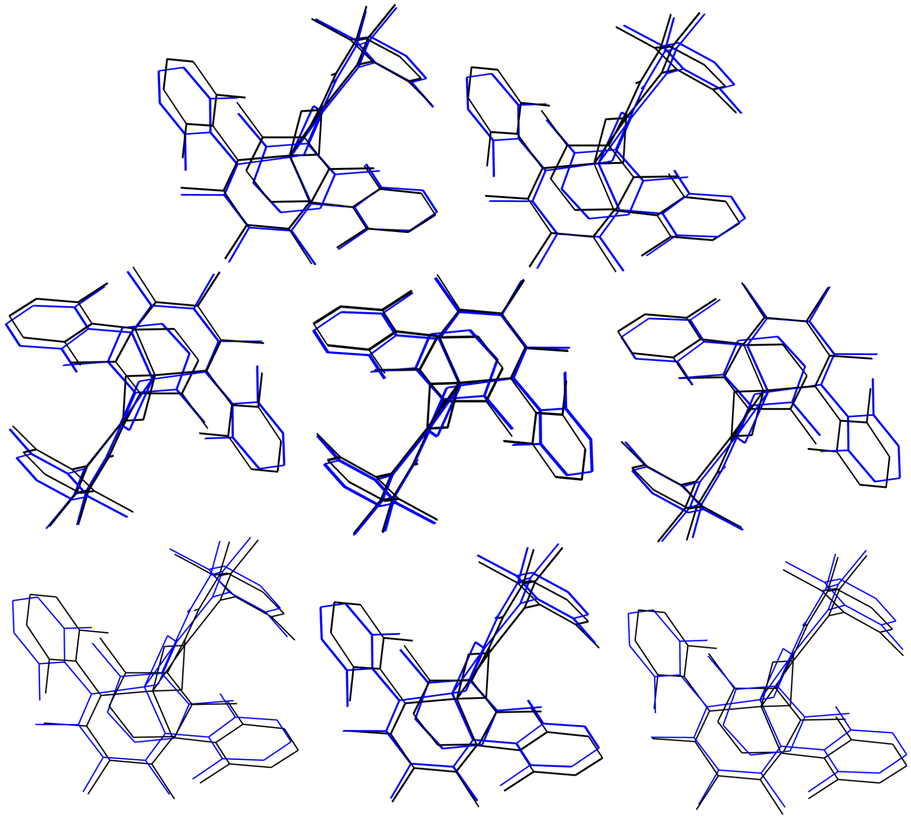

Figure S3. Overlay of the packing between **1** (black) and **1-LT** (blue). The RMSD between the packing motifs is 0.3067 Å; the similarity between the calculated powder diffraction patterns is 0.9973.

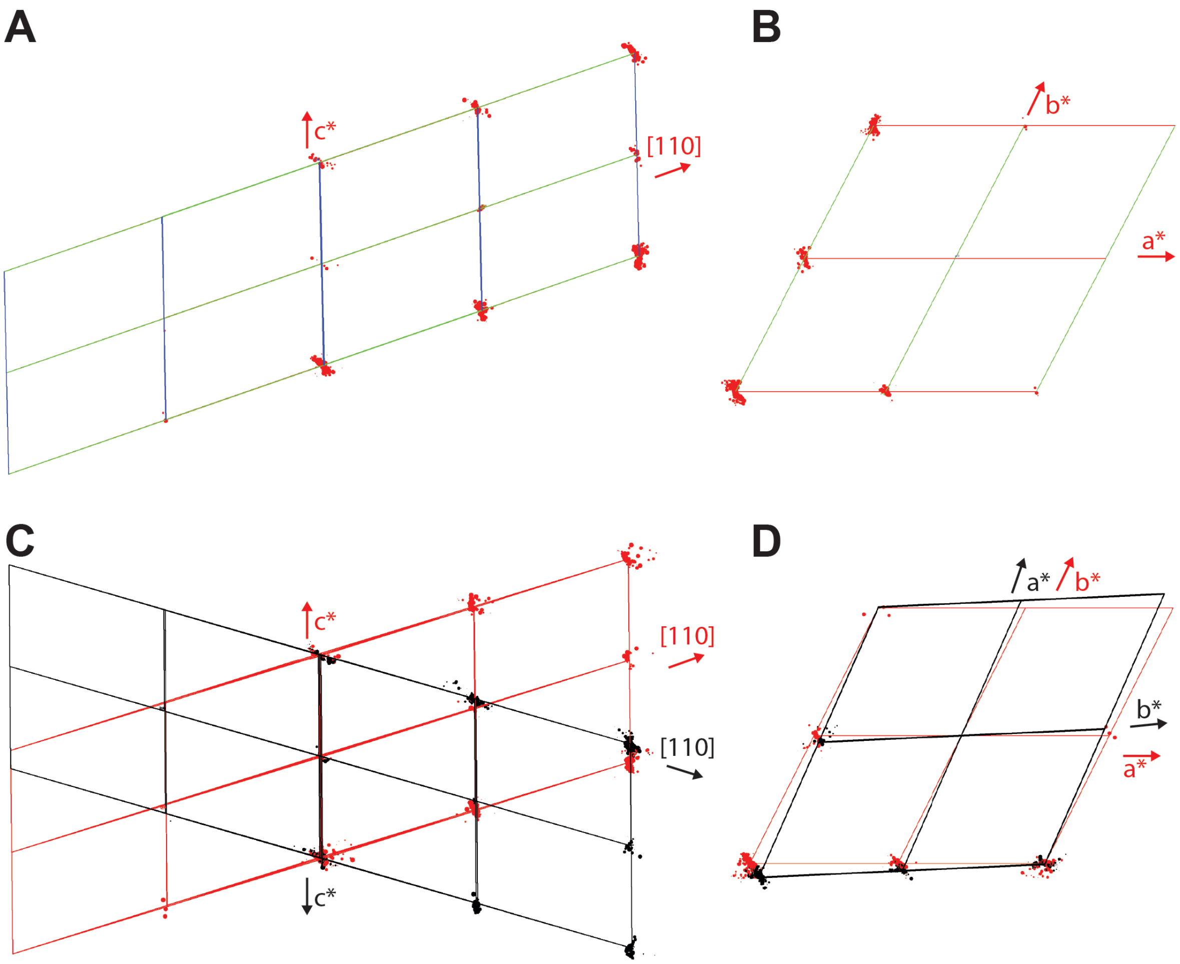

Figure S4. Comparison of the reciprocal lattices of **1** and **1-LT**. (A) View of **1** in *C*2/*c* where the diagonal vector between *a** and *b** is across the page, *c** is vertical. (B) View of **1** in *C*2/*c* where *a** is across the page, *b** is obliquely vertical. (C) View of the two twin domains in **1-LT i**ndexed in $P\bar{1}$. The diagonal vector between *a** and *b** is across the page, *c** is vertical. (D) View of **1-LT i**ndexed in $P\bar{1}$ in the *a***b** plane. The twinned domains are related by a 180° rotation about the [110] direction in reciprocal space. The twinned domains in C and D are distinguished by the red and black grids/spots.

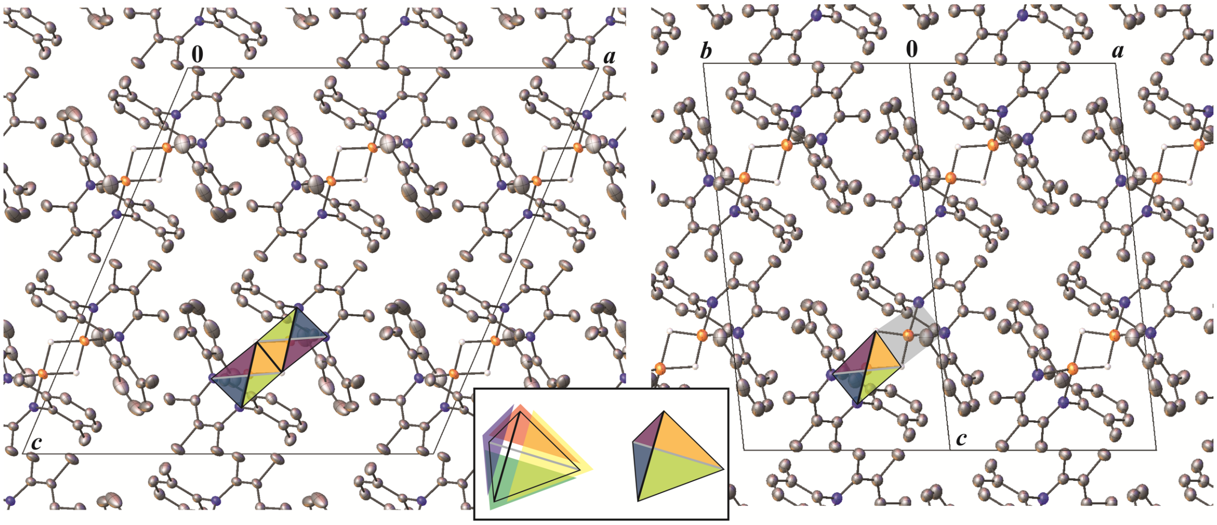

Figure S5. Comparison of unit cells between **1** (left; space group *C*2/*c*) and **1-LT** (right; space group $P\bar{1}$). The unit cell of **1** is displayed looking parallel to the *b* axis; **1-LT** is looking parallel to (110) face. The inset defines the faces of an arbitrary tetrahedron (left image has the colors slightly lifted from the tetrahedron faces; right has the colors on their respective faces). The same tetrahedron format is used to highlight the difference between the two unit cells. At 223 K, the 2-fold axis parallel to the *b* axis relates the two symmetry equivalent, pseudo-tetrahedral iron centers. As the sample is cooled to 93 K, nearly all of the crystallographic symmetry is destroyed. There is a loss of the 2_1 screw axis, the 2 rotation axis, and *c*-glides. Only half of the inversion centers are retained in the transformation from *C*2/*c* to $P\bar{1}$. The two iron centers are no longer related by the 2-fold rotation along the *b* axis. In the right image, one iron center is pseudo-tetrahedral; the other is pseudo-square planar, with the latter highlighted by the gray rectangle. Hydrogen atoms (except the hydrides) are omitted and thermal ellipsoids are shown at 25% for clarity.

Attempted Boltzmann fit of temperature-dependent Mössbauer data. The experimental zero field Mössbauer spectra of toluene-free crystals of the hydride dimer shown in Figure 2 are simulated in Figure S6 with a relaxation model for the electric quadrupole interaction in order to explain the collapse of quadrupole splitting from two distinct iron site with increasing temperature. For this model we assume correlated 'flipping' of the electronic environment of both ^{57}Fe-nuclei between two situations, 'T' and 'P', with two different electric field gradient (efg) tensors; the Mössbauer spectra for 'P' and 'T' at the two iron sites are indistinguishable. For the sake of simplicity, co-axial efgs of the same sign are assumed for 'T' and 'P', and cross-wise correlated 'hopping' between 'T' and 'P' for both iron sites, with a common rate that increases with temperature. In the spectra, the relaxation occurs then between pairs of (adjacent) quadrupole lines arising from 'P' and 'T'. (In case of opposite efg signs, pairs of the non-adjacent lines from the two doublets would coalesce.) Line shapes and positions of the relaxing pairs are calculated according to the Blume-Tjon model[3] for ^{57}Fe, and the efg-hopping model given by Litterst and Amthauer,[4] respectively. Similar expressions were derived previously by H. Wickman for paramagnetic relaxation.[5]

At 80 K, and practically also at 140 K, the system is in the static limit where two quadrupole doublets of equal absorption depth and Lorentzian line width are observed. At 140 K the isomer shifts and quadrupole splittings for both iron sites are slightly reduced as compared to 80 K (see Table S2 below). This is due to the second-order Doppler shift, and the presence of close lying excited spin-orbit states for the $3d^6$ configuration of iron(II).

In the range 140 – 170 K the coalescence processes of the two quadrupole doublets was simulated by the onset of quadrupole relaxation with temperature-dependent relaxation rates $1/\tau$, given in units of the Mössbauer line width of 0.2 mm/s (close to the natural line width). The same set of the other Mössbauer parameters is used throughout the simulations.

Table S2. Parameters from attempted quadrupole relaxation fit.

temperature	$\Delta E_{Q,1}$ (mm/s)	δ_1 (mm/s)	$\Delta E_{Q,2}$ (mm/s)	δ_2 (mm/s)	$1/\tau$, units of Γ [a]
80	0.74	0.60	2.16	0.43	0
140	0.71	0.54	1.95	0.43	0.09
150	"	"	"	"	0.17
160	"	"	"	"	0.69
170	"	"	"	"	10

[a] full width at half-maximum = 0.2 mm/s, corresponding to a frequency of ca. 2.3 MHz.

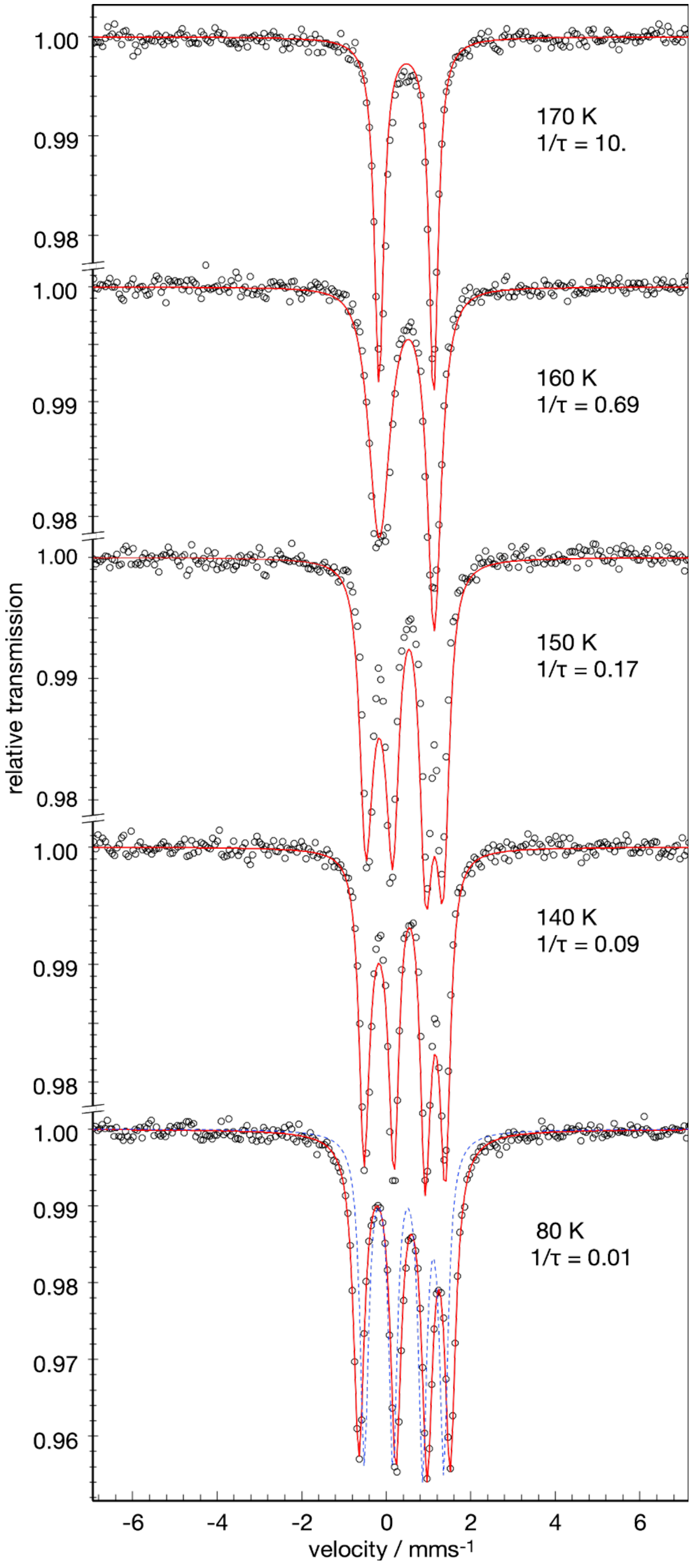

Figure S6. Fits to Mössbauer data using a quadrupole relaxation model with the Mössbauer parameters and rates given in Table S2. Note that the model was discarded because the rates (1/τ) yield an unrealistic Arrhenius plot (Figure S7) due to an unreasonably steep temperature dependence. The dotted blue line shows the simulation for 140 K superimposed on the 80 K spectrum, to enable comparison.

However, when the rates are placed on an Arrhenius plot (Figure S7), the best-fit line implies E_a = 10.4 kcal/mol and A = 2 x 10^{19} s^{-1}. The latter value is too high to be physically meaningful, and we conclude that the interconversion involves a cooperative process instead (see below).

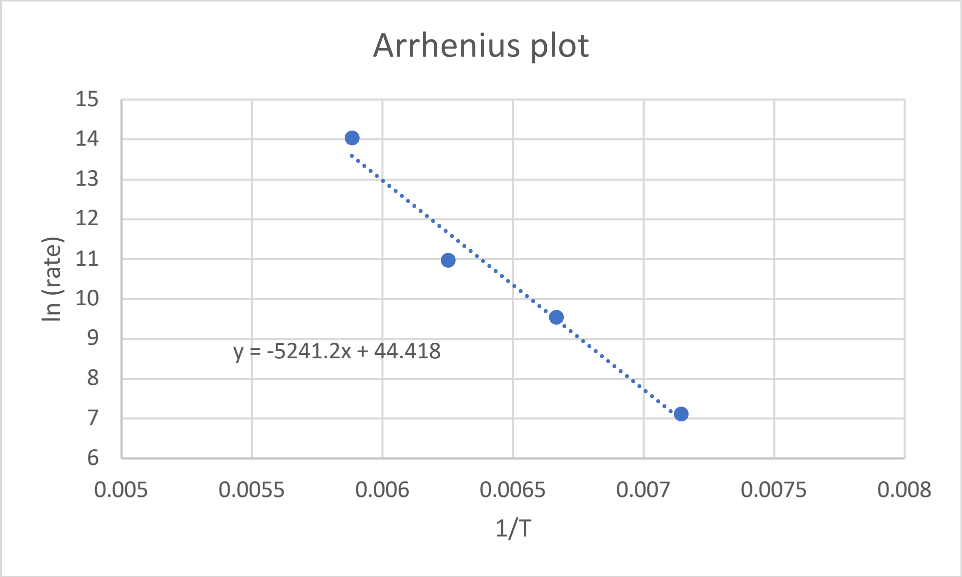

Figure S7. Arrhenius plot for the Boltzmann model.

Cooperative model for temperature-dependent Mössbauer data. The phase change observed for **1** at *ca.* 160 K is accompanied by a marked decoalescence of the Mössbauer spectrum from a single quadrupole doublet into two distinct subspectra (Figure 2 of main text). Table S3 summarizes the result of the fits for the spectral series with two Lorentzian quadrupole doublets of equal intensity; the temperature dependence of the quadrupole splitting for both subspectra is depicted in Figure S8 by symbols 'Δ' and '+'.

Table S3. Mössbauer data on **1** obtained from the fits with Lorentzian doublets of the zero-field spectra shown in Figure 2. Values are given in mm/s.

temperature	$\|\Delta E_{Q,1}\|$	δ_1	$\|\Delta E_{Q,2}\|$	δ_2
80	0.74	0.60	2.16	0.43
140	0.77	0.54	1.87	0.43
150	0.85	0.53	1.75	0.43
160	1.03	0.50	1.48	0.45
170	1.25	0.47	1.25	0.47

[a] Lorentzian full width at half-maximum.

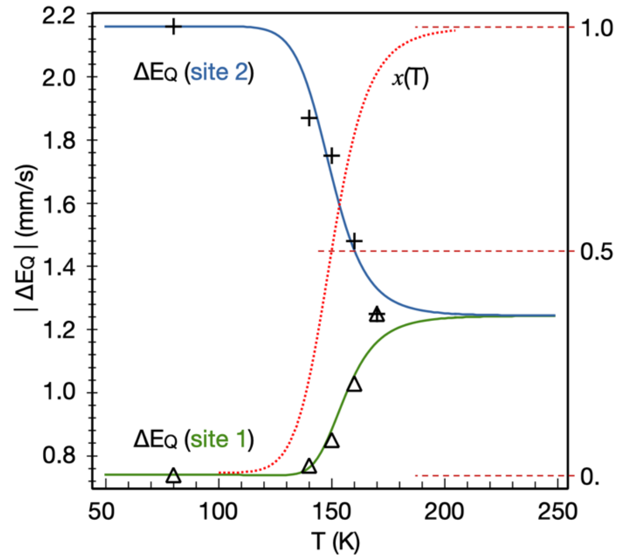

Figure S8. Temperature dependence of the quadrupole splitting (left axis) found for sites 1 (symbol Δ) and 2 (symbol +). The green and blue lines are simulations for a phase transition by using the Sorai-Seki domain model to describe the fraction $x(T)$ of molecules in the high-temperature phase (red curve and right axis).

Coalescence of Mössbauer spectra arises from dynamic processes and has been observed in the disorder of O_2 bound to iron porphyrin complexes,[6] or the Jahn-Teller distortion of the cubic iron(II) site in cesium iron phosphate hexahydrate. In the latter case, time averaging of the distortion over the three canonical axes gives pseudo-cubic symmetry and collapses the otherwise large quadrupole splitting of the high-spin iron(II) ions to a single doublet above 100 K without significant line broadening.[7]

In the following model, the coalescence of the Mössbauer spectra of **1** arises from a phase transition from a static low-temperature to a dynamic high-temperature phase, the latter having fast and concerted interconversion of the electronic environment of the two iron sites. For Mössbauer spectra the relevant time scale for fast processes is *ca.* 10^{-7} s. In our model, we adopt two limiting *EFG*(I) and *EFG*(II) for the two iron sites. At low temperatures (< 100 K), iron *site 1* may give rise to *EFG*(I) at the ^{57}Fe nucleus, whereas *site 2* exhibits *EFG*(II), or vice versa. The EFGs correspond to the two distinct quadrupole splittings observed at 80 K. Initially occurring flips of the dimer configuration are assumed to be slow. The onset of the phase transition at higher temperatures enables an increasing fraction $x(T)$ of molecules to swap the dimer configuration easily and quickly such that for short time slices in our example *site 1* exhibits *EFG*(II) and *site 2* has *EFG*(I). Note, if this 'hopping' of the cluster configuration were to be slow on the Mössbauer time scale (ca. 10^{-7} s), nothing would change in the spectra because the iron sites *per se* are indistinguishable in this model. However, if the flips occur at fast rates, the quadrupole splitting for each site is determined by the time-average of *EFG*(I) and *EFG*(II), depending on weighting factors *w*(I) and *w*(II) for *EFG*(I) and *EFG*(II):

$$EFG_1 = w(\mathrm{I}) * EFG(\mathrm{I}) + w(\mathrm{II}) * EFG(\mathrm{II}) \quad (1)$$

$$EFG_2 = w(\mathrm{I}) * EFG(\mathrm{II}) + w(\mathrm{II}) * EFG(\mathrm{I}) \quad (2)$$

Moreover, the swaps of the dimer configurations cannot be stationary but must 'hop around' the ensemble, also on a fast time scale, since no low-temperature fraction of subspectra coexists with the coalescence spectra. Due to the time and ensemble average, the weight factors are given by the temperature-dependent fraction $x(T)$ of molecules in the high-temperature phase

$$w(\mathrm{I}) = 1 - 0.5 * x(T) \quad (3)$$

$$w(\mathrm{II}) = 0.5 * x(T) \quad (4)$$

Here, the factor 0.5 takes into account the 50:50 average of both electronic environments for both iron sites in the high temperature limit.

Since the coalescence of the quadrupole spectra occurs within a temperature range of less than 40 K, a Boltzmann-driven process of the Arrhenius-type is excluded (see above for our unsuccessful attempt to fit this model). Thus this second model involves cooperativity and an entropy-driven phase transition occurring within domains. As shown in Figure S8, a good fit of the quadrupole splittings was possible with the Sorai-Seki domain model[8,9] describing the fraction of molecules $x(T)$ in the high-temperature phase

$$x(T) = 1 / [1 + \exp\{(n\Delta H/R)(1/T - 1/T_c)\}], \qquad (5)$$

where T_c is the transition temperature, R is the gas constant and $n\Delta H$ is an enthalpy factor that depends on the size n of the domains, and which determines the width of the transition. Least-squares optimization yielded the transition temperature $T_c = 150$ K, and an enthalpy factor, $n\Delta H = 1525$ cm^{-1}.

In accordance with the magnetic Mössbauer spectra described in the main text, the quadrupole splitting due to EFG(I) was taken to be negative, $\Delta E_{Q1} = -0.74$ mm/s whereas ΔE_{Q2} was taken to be positive. Moreover, the asymmetry parameters η of the electric field gradients were taken to be zero. With this choice, the average quadrupole splitting derived from the 80 K values, $(\Delta E_{Q1} + \Delta E_{Q2})/2 = 0.74$ mm/s (if ΔE_{Q1} is negative) is far from the coalescence value $\Delta E_{Q1,2} = 1.25$ mm/s at high temperature. This suggests that we cannot simply average the quadrupole splittings, because the *EFG*(I) and *EFG*(I) tensors are not collinear. This agrees with the fits to the magnetic Mössbauer spectra, the SQUID data, and the computational results.

Note that quadrupole splittings in general are not additive, but electric field gradients are. This holds also here for the superposition of field gradients in a hopping or other coalescence process. Before addition of *EFG*s, the components V_{ij} of both tensors have to be expressed in a common reference frame, and the result has to be diagonalized to determine the new quadrupole splitting. Since field gradients are traceless, $V_{xx} + V_{yy} + V_{zz} = 0$, the V_{xx} and V_{yy} components have signs opposite to the main component V_{zz}, which determines the quadrupole splitting. Therefore an Euler rotation of the electric field gradient tensor *EFG*(II) with respect to the principal axis system of *EFG*(I) was essential for correct superposition of EFGs at high and intermediate temperatures and the proper simulations shown in Figure S8 (green and blue lines). The best fit for a rotation around the y-axis yielded an Euler angle $\beta = 110°$. We note that this solution may be not unique since consideration of the asymmetry parameters η would lead to a variety of possible solutions that cannot be disentangled from the limited number of data available. For the sake of simplicity we kept η at zero for both sites and limited the Euler

rotations to the angle β. Interestingly, the angle β = 110° is close to the value found independently from the magnetic Mössbauer spectra (β = 95°, see below).

In summary, the (de)coalescence of the quadrupole spectra of **1** at 150 K can be well described as the result of a phase transition in the solid material which leads to fast 'hopping' of the electronic environments of sites 1 and 2 in the dimer with rates $>10^7$ s^{-1}. Locally, the dynamics causes superposition and time-averaging of two different electric field gradients *EFG*(I) and *EFG*(II) with different weight factors at sites 1 and 2. The Sorai-Seki domain model for the phase transition yielded a good fit of the coalescence behavior.

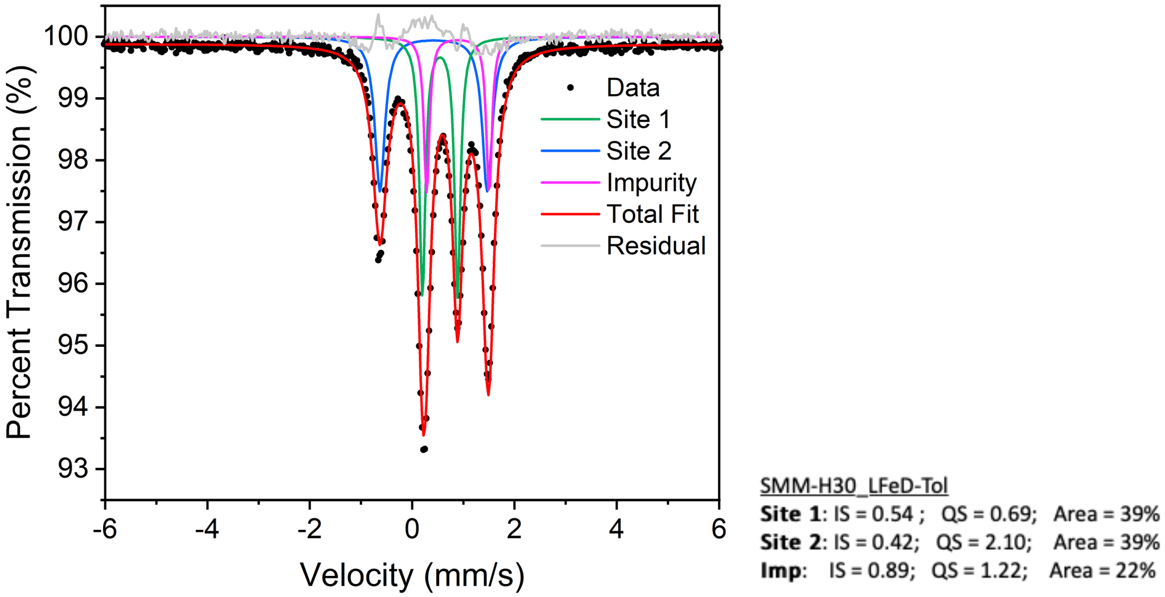

Figure S9. Zero-field Mössbauer spectrum (80 K) of crystals from toluene, from the sample used for neutron diffraction data. The data are shown as black circles, the fits are shown with colored lines, and the residual (fit - data) is shown as a light grey line. The fit required the inclusion of an impurity (pink line), which likely arises from some sample exposure.

The fit parameters of the sites are similar to those for **1-LT** (**site 1:** IS = 0.60, QS = 0.74; **site 2:** IS = 0.43, QS = 2.16) at the same temperature.

X-ray crystal structure of 1-toluene. Low-temperature diffraction data (ω-scans) were collected at the Advanced Light Source, Lawrence Berkeley National Laboratory, on a Bruker D8 goniometer coupled to a PHOTON 100 detector with synchrotron radiation (λ = 0.7749 Å). The data were integrated with the APEX2 software package and absorption corrections were applied with SADABS.[10] The structure was solved with SHELXT and was refined against F^2 on all data by full-matrix least squares with SHELXL.[11] All non-hydrogen atoms were refined anisotropically. Most hydrogen atoms were included in the model at calculated positions and refined using a riding model. The isotropic displacement parameters of all hydrogen atoms were fixed to 1.2 times the *U* value of the atoms to which they are linked (1.5 times for methyl groups). The only exceptions are H1 and H2, which were found in the difference map and freely refined. One reflection was recorded improperly due to instrument artifacts and subsequently omitted. The absolute structure parameter was 0.028(6), indicating that the correct enantiomer was used. The full numbering scheme of **1-toluene** can be found in the CIF file. CCDC number 1940223 contains the supplementary crystallographic data.

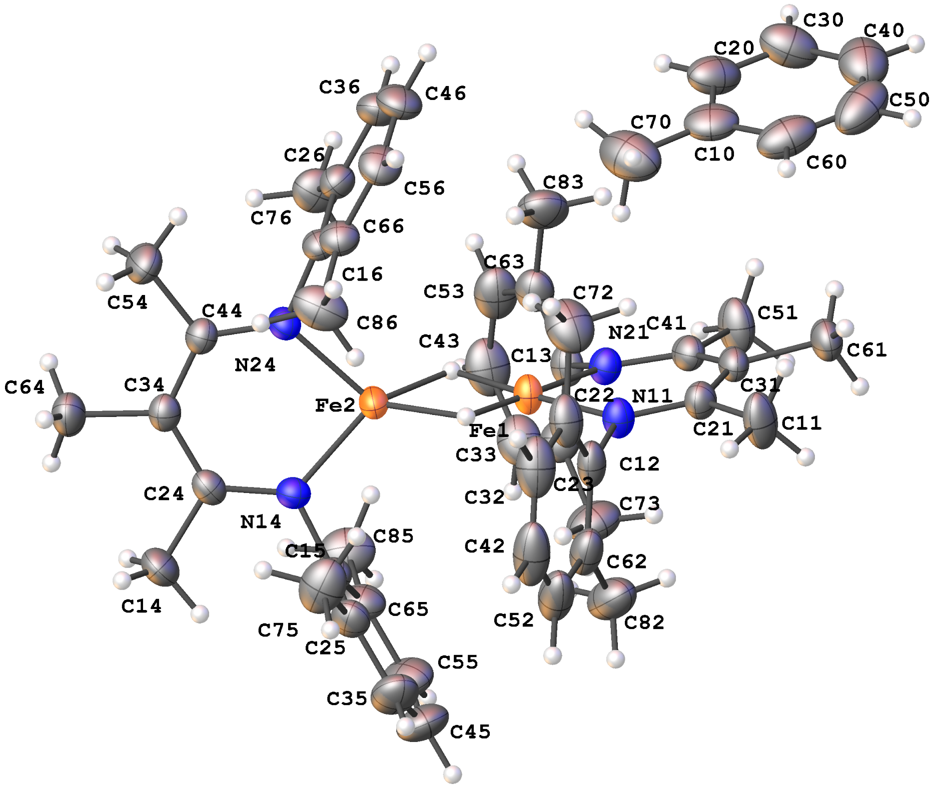

Figure S10. A partial numbering scheme of **1-toluene** with 50% thermal ellipsoid probability levels. The hydrogen atoms are shown as circles for clarity.

Neutron structures of 1-H and 1-D. Data for **1-H-neut** and **1-D-neut** were collected on the TOPAZ single-crystal time-of-flight (TOF) Laue diffractometer at the Spallation Neutron Source, Oak Ridge National Laboratory.[12] A plate-shaped crystal of **1-H**, with dimensions of 2.9 x 0.8 x 0.75 mm, was mounted on the tip of a MiTeGen loop using Krytox grease, and transferred to the TOPAZ goniometer for data collection at 100 K. A similar approach was used for **1-D-neut**, with dimensions 2.04 x 0.72 x 0.70 mm. The integrated raw Bragg intensities were obtained using the 3-D ellipsoidal Q-space integration in accordance with previously reported methods.[13] Data reduction, including neutron TOF spectrum Lorentz and detector efficiency corrections, was carried out with the ANVRED3 program.[14] A Gaussian numerical absorption correction was applied. The reduced data were saved in the SHELX HKLF2 format, where the wavelength is recorded separately for each individual reflection, and were not merged. The initial neutron structure was refined starting from the X-ray structure, with fixed positions of non-hydrogen atoms using SHELXL-2014.[11] The positions of hydride atoms in **1-H-neut** were located in difference-Fourier maps calculated using the neutron data. For **1-D-neut,** the final model suggested that it coexists with the hydride complex. To refine the site occupancy of hydrogen at the deuteride site in the crystal structure, a scale factor for the atomic displacement parameters (APDs) was first applied to account for the zero-point motion of hydrogen. The ADPs for terminal H and D atoms could be constrained such that ADP(H) = $\sqrt{2}$ADP(D).[15] However, the factor is expected to be smaller for bridging hydride due to the difference in reduced mass for Fe-H-Fe and Fe-D-Fe.

We calculated the experimental ADP scale factor for the bridging hydrides from the ratio of the averaged values of principal U_{ii} components of the ADPs in **1-H-neut** and **1-D-neut**, assuming the latter is fully deuterated. A scale factor of 1.32 for the H/D ADPs was obtained and used to scale the hydride displacement parameters for **1-D-neut** in the subsequent refinement of the neutron structure. The ADPs of the deuterides were allowed to vary, while those of hydrides were constrained to be 1.32 times that of the deuterides. The positions of the H/D atoms are constrained to be identical, and their overall site occupancy was constrained to 1. In the final refinement, the D/H ratio converged to 0.93(2)/0.07(2).

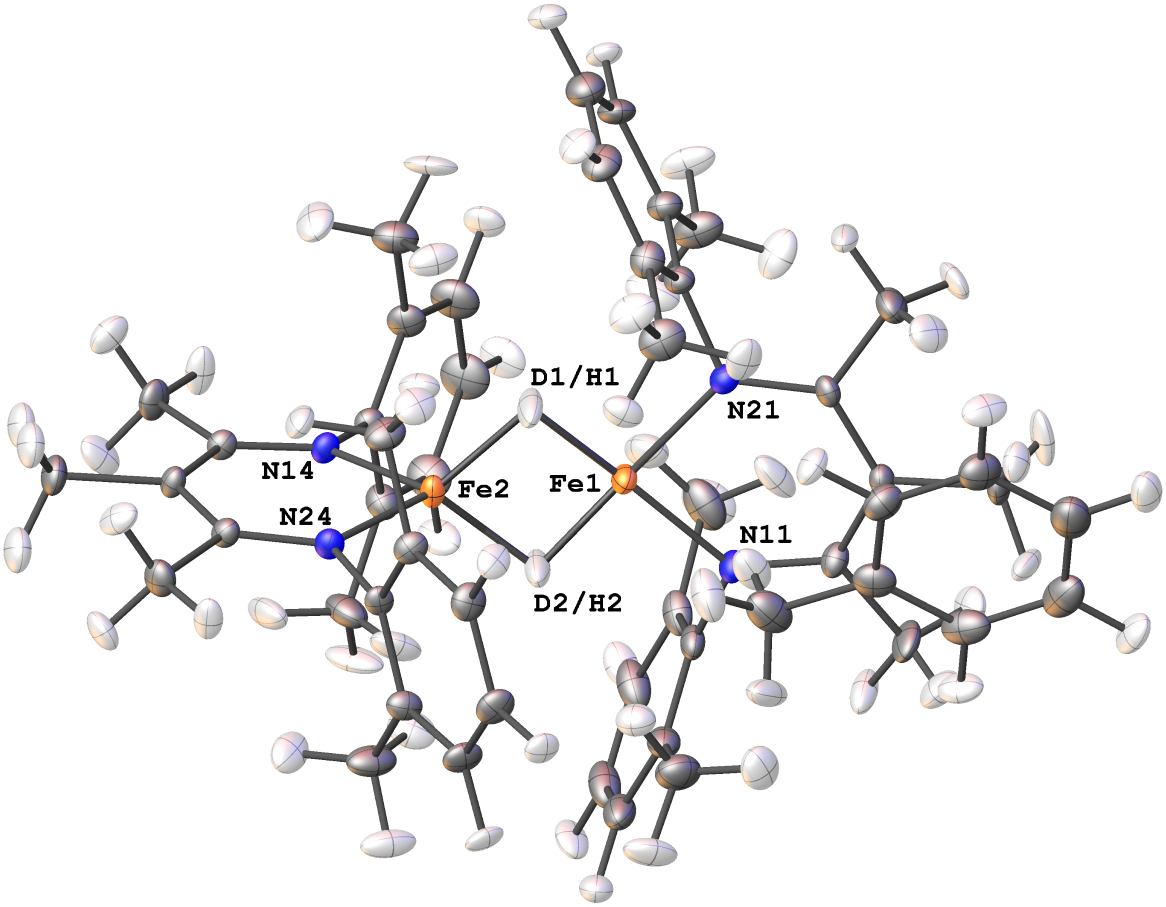

Figure S11. A partial numbering scheme of **1-D-neut** with 50% thermal ellipsoid probability levels. The hydrogen and the bridging deuterium atoms are shown with 50% thermal ellipsoid probability levels where applicable. As noted above, the bridging D atoms are modeled as 93% deuterium and 7% protium; this ratio is revealed by the data because of the different neutron scattering from ^{1}H vs. ^{2}H nuclei.

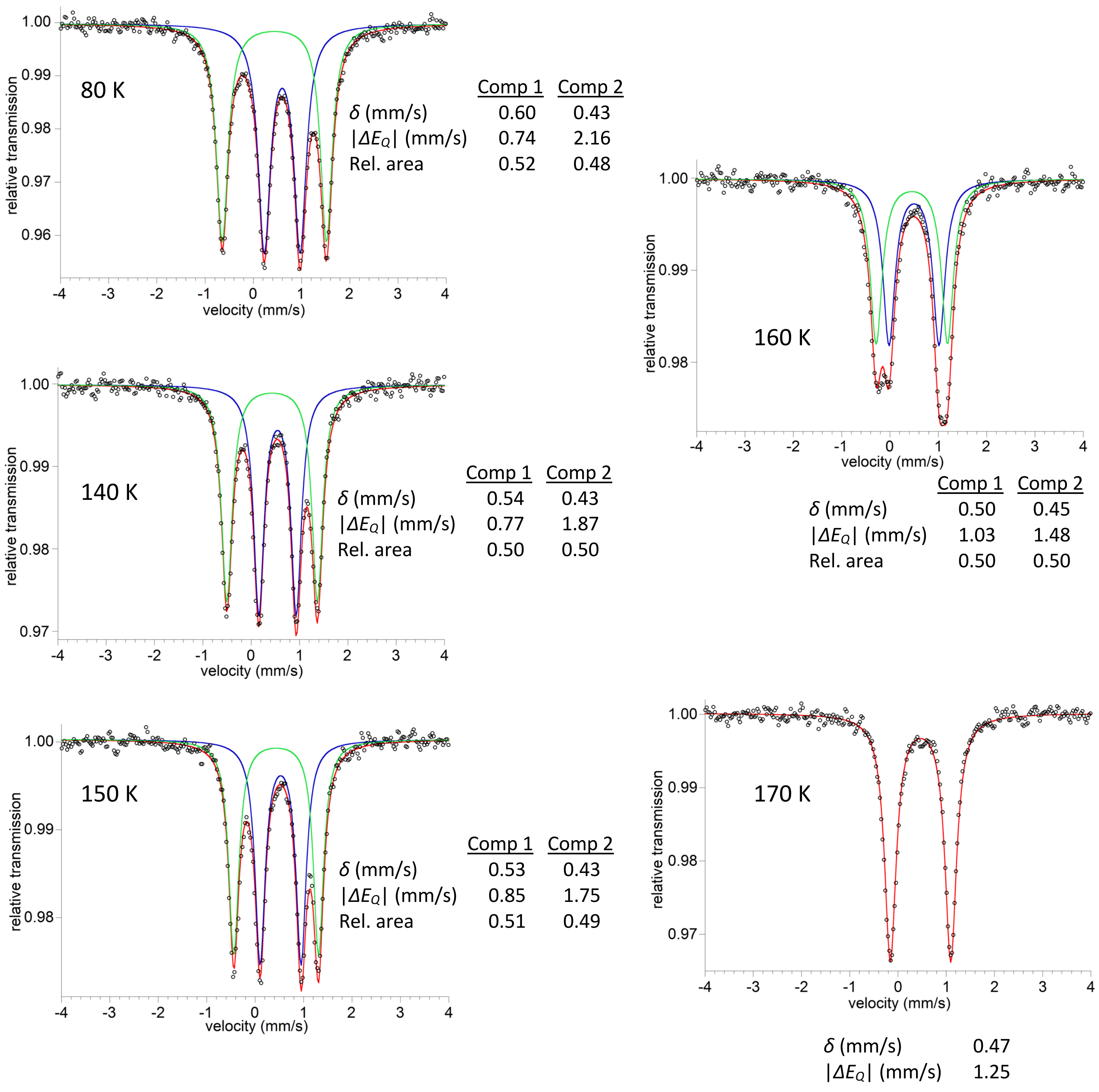

Figure S12. Zero-field Mössbauer spectra of **1-D** at various temperatures. The isomer shifts are plotted in Figure S13.

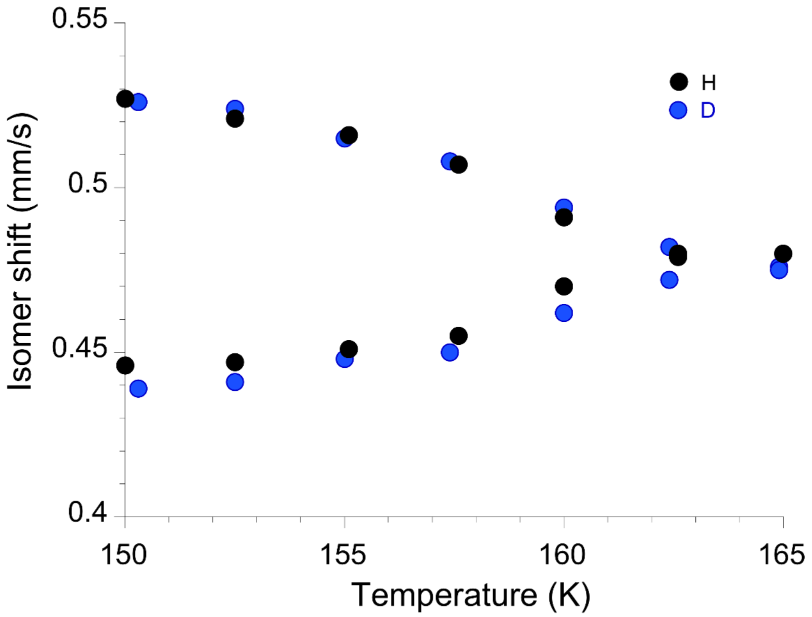

Figure S13. Plot of the isomer shifts of the two peaks as a function of temperature, from Mössbauer spectra collected with closely spaced temperatures. This shows that the coalescence temperature of 163 ± 3 K is not significantly different between H and D isotopologues.

Magnetic susceptibility data were measured on a powder sample (KCM-II-123) in the temperature range 2 - 290 K by using a SQUID magnetometer with a field of 1.0 T (MPMS-7, Quantum Design, calibrated with standard palladium reference sample, error <2%). Multiple-field variable-temperature magnetization measurements were done at 1 T, 4 T, and 7 T in the range 2-260 K with the magnetization equidistantly sampled on a 1/T temperature scale. Samples were flame-sealed in NMR tubes and fixed with a plug of quartz-wool since we could not embed the sensitive compound in a wax. A corresponding compensation tube was added to balance the holder arrangement in a straw. The SQUID response curves (raw data) have been corrected for holder contributions by subtracting the corresponding response curves obtained from separate measurements without sample material. The experimental magnetization data were obtained from independent simulation of the corrected SQUID response curves, and subsequently were corrected for underlying diamagnetism by use of tabulated Pascal's constants, as well as for temperature-independent paramagnetism. Handling and simulation of the SQUID raw data as well as spin-Hamiltonian simulation of the susceptibility and magnetization data were done with our own package julX.SL for exchange-coupled systems.

Magnetic Susceptibility and magnetic Mössbauer spectra were simulated with the program *mx.SL* (by E.B.) by diagonalization of the spin Hamiltonian for one or two exchange-coupled electronic spins S_i

$$H = -2J\vec{S}_{Td}\cdot\vec{S}_{SP} + \mu_B\vec{B}\left(\tilde{g}_{Td}\vec{S}_{Td} + \tilde{g}_{SP}\vec{S}_{SP}\right) + \sum_{i=Td,SP} D_i\left[S_{z,i}^2 - {}^{1}/_{3}\, S_i(S_i+1) + E/D\,(S_{x,i}^2 - S_{y,i}^2)\right] \quad (6)$$

where the first term is the Heisenberg-Dirac-van Vleck Hamiltonian with the exchange coupling constant *J*, the *g* symbols denote the local electronic *g* matrices, and *D* and *E/D* are the single-ion axial and rhombic zero-field splitting (*zfs*) parameters for the pseudo tetrahedral

(Td) and square planar (SP) sites. The hyperfine interaction for ^{57}Fe on Td and SP sites was calculated with the usual nuclear Hamiltonian

$$\hat{H}_{nuc} = \hat{\vec{I}} \cdot \overline{\overline{A}} \cdot \hat{\vec{S}} - g_N \mu_N \hat{\vec{I}} \cdot \vec{B} + \hat{H}_Q \tag{7}$$

where A is the hyperfine coupling matrix and S denotes the local spin of Td and SP sites. H_Q is the usual Hamiltonian for electric quadrupole interaction. Isomer shifts were taken to be additive.

Details on Magnetic Susceptibility. We were able to fit the data with different spin Hamiltonian (SH) models (eq. S6 above), one with two $S = 2$ subsites (S2S2 model) and one with an $S = 2$ subsite and an $S = 1$ subsite (S2S1 model, shown in Figure 7 of main text).

The S2S2 model with two equivalent $S = 2$ subsites required $g = 2.0 – 2.2$, large negative zero-field splitting (zfs) with D of $–52$ cm^{-1}, and exchange coupling of $J = +3.9$ cm^{-1} (Figure S14). Importantly, this fit required us to postulate the presence of a diamagnetic impurity comprising 15% of the sample (to match in particular the high-temperature level $\chi_M T(T)$ without unreasonably low g-values below 2), despite the lack of evidence for such an impurity in the samples from ^{1}H NMR or Mössbauer spectroscopy. Moreover, the equivalent spin states of the S2S2 model do not agree with the differing Mössbauer parameters of the two iron sites, which have very different isomer shifts in spite of identical coordination numbers and types of ligands.

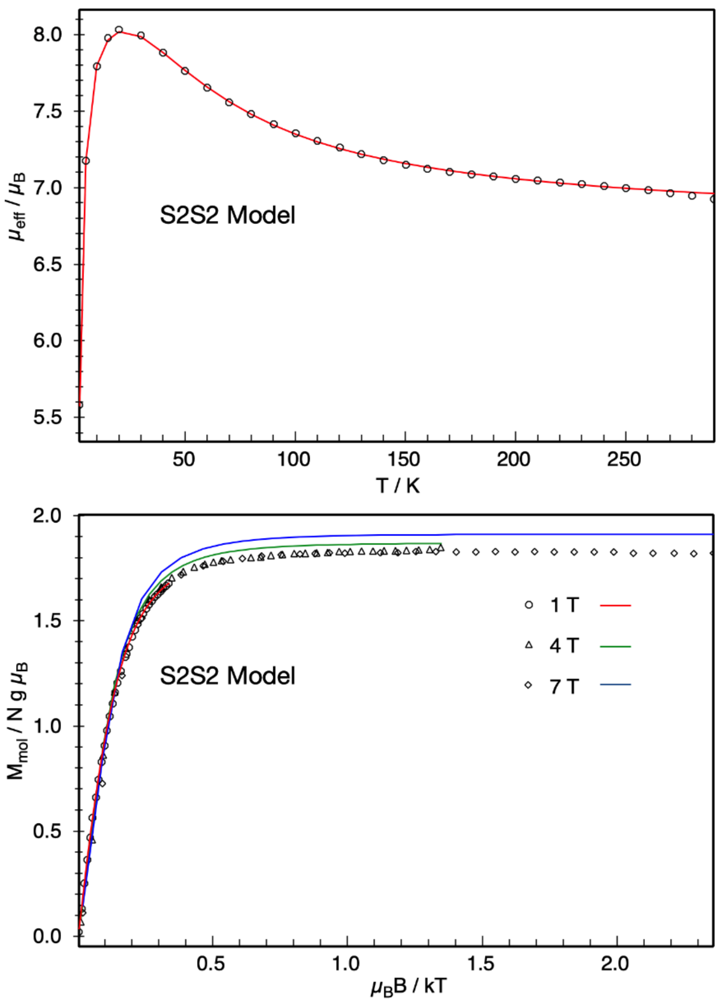

Figure S14. Top: Temperature dependence of the effective magnetic moment of **1** recorded with 1 T applied field. Bottom: 1/T dependence of the iso-field magnetization recorded with fields of 1, 4 and 7 T. The experimental data were corrected for a TIP-like contribution to χ of 200 x 10^{-6} emu. The colored lines represent a global SH simulation using the **S2S2 model** with S_{Td} = 2, S_{SP} = 2 and the following SH parameters: $J(\mathrm{Td}-\mathrm{SP}) = +3.9\ \mathrm{cm}^{-1}$, $g_{Td} = g_{SP} = (2.07, 2.07, 2.15)$, $D_{Td} = D_{SP} = -52.1\ \mathrm{cm}^{-1}, \frac{E}{D} = 0$. Both sites, Fe_{Td} and Fe_{SP} have identical spin-Hamiltonian parameters, but a 14.6 % impurity with S = 0 had to be included in the model.

As described in the text and Figure 7, we also tested a S2S1 model with one S = 2 subsite and one S = 1 subsite. The S2S1 model did not require impurities to fit the data, though it requires anisotropic g-values that are significantly shifted from 2 at both the pseudo-tetrahedral (Fe_{Td}) and square-planar (Fe_{SP}) iron sites, with distinctly different zfs parameters. We were able to model the $\chi_M T(T)$ data as well as the multi-field $M(\mu_B B/kT)$ data well with the following SH parameters: S_{Td} = 2, g_{Td} = 2.47, 2.47, 2.61, and D_{Td} = –26 cm^{-1}, and S_{SP} = 1, g_{SP} = 3.65, 3.65,

1.0, and D_{SP} = +40 cm^{-1}. The exchange coupling constant was J = +63 cm^{-1} (using the convention $-2J\ S_{Td}{\cdot}S_{SP}$). We restrained the local D and g matrices to be axial to avoid overparametrization of the model. However, for fitting the D_{SP} tensor and g_{SP} matrix of the Fe_{SP} site had to be rotated relative to the magnetic axes of the Fe_{Td} site by an Euler angle β = 90(±10)° around the y axis. The rotation makes the local magnetic quantization axes, z_{SP} and z_{Td}, of SP and Td sites perpendicular to each other, and x_{SP} to be along the z_{Td} direction (Figure 8, top). These SH axes could be associated with the molecular structure of **1** as indicated in Figure 8, wherein the idealized four-fold axis of the Fe_{SP} site perpendicular to its ligand plane is required to be the principal z axis of the local $\boldsymbol{g}_{SP}$- and $\boldsymbol{D}_{SP}$- matrices, whereas the pseudo-S_4 axis for the Fe_{Td} site along the Fe-Fe vector is the principal quantization axis for the corresponding matrices $\boldsymbol{g}_{Td}$ and $\boldsymbol{D}_{Td}$ for the Td site.

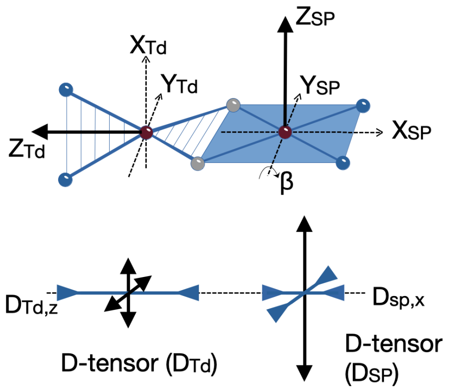

Figure 8 (reproduced from main text for convenience). Top: Schematic view of the rotated magnetic axes for the square planar (SP) relative to the tetrahedral (Td) sites of **1** as obtained from the simulation of the magnetic data. Bottom: Relative orientation of the ***D***-tensors for Td and SP sites. The double arrows pointing outward and inward indicate the positive and negative tensor components, respectively. Note that the ***D***-tensors are traceless in the SH formalism, *i.e.* the total length of positive and negative double arrows is zero. Analogous plots would hold for g-matrices, electric field gradient tensors (V_{ij}), and magnetic hyperfine coupling tensors $\boldsymbol{A}$ for ^{57}Fe. All local matrices for Td and SP sites, respectively, have been kept essentially collinear (see Mössbauer section below).

Diagonalization of the spin Hamiltonian (eq. S6) with the parameters given above yields three reasonably well separated m_s-manifolds: a septet ground state (S_{total} = 3), and a quintet and triplet excited states as expected for ferromagnetic coupling of S_{Td} = 2 and S_{SP} = 1 (Figure S18 below). Although there is a relatively large uncertainty of J (est. ±20 cm^{-1}) for the parameter subspace of tensor rotations, the low-temperature magnetic properties of **1** can be interpreted by approximating it as a fully separated S_{total} = 3 manifold. Spin projection arguments show that the S_{Td} = 2 site provides the leading contribution to the properties of this dimer ground state. In the hypothetical limit of infinitely strong ferromagnetic coupling of S_{Td} = 2 and S_{SP} = 1 sites, the spin projection coefficients[16] for zfs and g-values of the septet are dominated by $\boldsymbol{D}_{Td}$ and g_{Td}: $D_{total} = (2/5)\ \boldsymbol{D}_{Td} + (2/30)\ \boldsymbol{D}_{SP}$ and $g_{total} = (2/3)\ g_{Td} + (1/3)\ g_{SP}$. Therefore, the negative zfs parameter D_{Td} = -26 cm^{-1} for the Td site is the main origin of the magnetic anisotropy in **1**.

Interestingly, in spite of the low projection factor (2/30) for the SP site, the rotated $\boldsymbol{D}_{SP}$ tensor was also important for a satisfying global fit of χ_MT(T) as well as the multi-field $M(\mu_B B/kT)$ data. In the sense of a perturbation contribution, the negative *xx* and *yy* components of $\boldsymbol{D}_{SP}$ being aligned by the Euler rotation along the negative main component of $\boldsymbol{D}_{Td}$ (shown as blue double-arrows in Figure 8) helped to sufficiently isolate the low-lying pair of highest m_s levels and therefore to foil nesting of the simulated magnetization curves at different fields.

The large zfs of the S_{total} = 3 ground state, being dominated by axial D_{Td} = -26 cm^{-1}, causes the Zeeman splitting of the low-lying "$m_{s,total}$ = ±3" levels to be strong for fields in the z-direction but weak in x/y directions. This feature renders an "easy axis of magnetization." For **1**, the magnetic anisotropy is increased by g anisotropy of the ground state septet (Figure S16), given by the large $g_{Td,z}$ value for the Td site, and enforced by the contribution of the (rotated) g_{SP} matrix. The alignment of $\boldsymbol{g}_{Td}$ and $\boldsymbol{g}_{SP}$ matrices, being collinear with the local $\boldsymbol{D}$-tensors sketched in Figure 8, helped the fit to reproduce the experimental over-shooting of $\chi_M T(T)$ around 20 K. The large g components along z_{Td} strengthen the magnetization of the (z-polarizable) high m_s levels, which are exclusively populated at low temperatures. In contrast,

lower g_{Td} values in the *x/y* direction reduce the magnetization of the other levels with lower m_s that become considerably populated above 20 K. This difference in magnetization of the m_s levels causes the observed overshooting of $\chi_M T(T)$ with temperature, rather than the more typical assignment of overshooting to ferromagnetic coupling. The same behavior was previously observed for an $S = 1$ $\{FeNO\}^8$ species[17] and a two-coordinate $S = 2$ imidoiron(II) species,[18] both of which feature large, negative D and g_z larger than 2.

Large and anisotropic shifts in *g* values have been observed previously in $S = 1$ square-planar iron(II) compounds, such as $[Fe^{II}(TPP)]$, which possesses highly anisotropic *g* values ($g_{x,y} = 3.0$ and $g_z = 1.7$; $g_{iso} = 2.6$), as well as unusually large and positive D of $+94\ cm^{-1}$.[19] The qualitative orbital diagram is characterized by near degeneracy of *three* close-lying orbital states that are essentially $^3A_{2g}$ and ^3E_g in nature and correspond to $(d_{xy})^2(d_{z2})^2(d_{xz,yz})^2$ and $(d_{xy})^2(d_{xz,yz})^3(d_{z2})^1$ / $(d_{z2})^2(d_{xy})^1(d_{xz,yz})^3$ electron configurations of iron(II) in SP geometry.

Magnetic Mössbauer Spectroscopy. The magnetic properties of **1** indicate ferromagnetic coupling of $S_{Td} = 2$ and $S_{SP} = 1$ and negative zfs of the ground state manifold with two low-lying high-m_s substates, due to the combined effect of negative D for the Fe_{Td} site and rotated positive $\boldsymbol{D}_{SP}$ tensor for the Fe_{SP} site. We tested the consistency of this description through simulation of magnetic Mössbauer spectra. The spectrum recorded at 4.2 K with an applied field of 4 T perpendicular to the γ rays (Figure S15) shows a well resolved hyperfine pattern that could be simulated by using the spin Hamiltonian parameters taken from the magnetic susceptibility data and the electric hyperfine parameters from the 80 K zero-field spectrum, modulated by tiny increases of the isomer shifts to 0.65 and 0.47 mm/s, and quadrupole splittings 0.82 and 2.25 mm/s for Fe_{Td} and Fe_{SP} sites, respectively. Ferromagnetic coupling of the Fe_{Td} and Fe_{SP} sites in **1** was needed in the SH models to fit the applied field Mössbauer spectra, but the actual strength of the spin coupling cannot be well determined from the Mössbauer data. The magnetic hyperfine coupling constants along the easy (*z*) axis of the system fit to values $A_{Td,zz}/g_n\beta_n = +13.2$ T and $A_{SP,xx}/g_n\beta_n = +58.7$ T (with the $\boldsymbol{A}_{SP}$ tensor being

rotated like $\boldsymbol{D}_{SP}$ and $\boldsymbol{g}_{SP}$ as shown in Figure 8 such that $A_{SP,xx}$ is oriented along the easy magnetic (z_{Td}) axis of the ground state.) In contrast, the other A components cannot be determined because of the vanishing electronic spin expectation values in x and y directions. The positive A values indicate the presence of a strong orbital contribution to the A tensor. With regard to the Fe_{SP} site, this is consistent with the strong positive transverse $A_{\perp}$ value found for [Fe^{II}(TPP)], and in our case we only sense one component of the A tensor, namely the one pointing along the easy axis of the Fe_{Td} site.

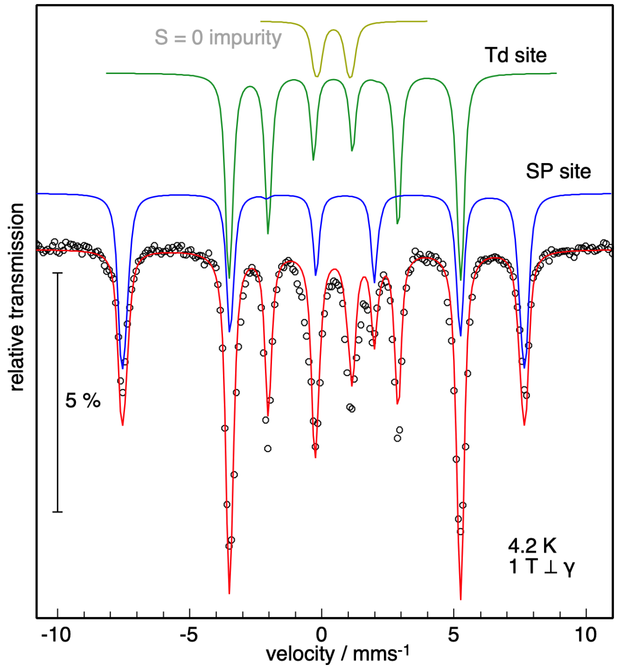

Figure S15. Magnetic Mössbauer spectrum of **1** recorded at 4.2 K with a field of 1 T applied perpendicular to the γ rays. The colored lines represent a SH simulation (eq. S6-S7) using the S2S1 model with the electronic SH parameters as given in Figure 7, and with hyperfine parameters as described above. The values for δ and ΔE_Q were adopted from the zero-field Mössbauer spectra recorded at 80 K. Green line: subspectrum of the Fe_{Td} site, blue: subspectrum of the Fe_{SP} site, red: 1:1 superposition of subspectra, including a minor 8% contribution from a diamagnetic impurity (δ = 0.44 mm/s, and ΔE_Q = 1.25 mm/s).

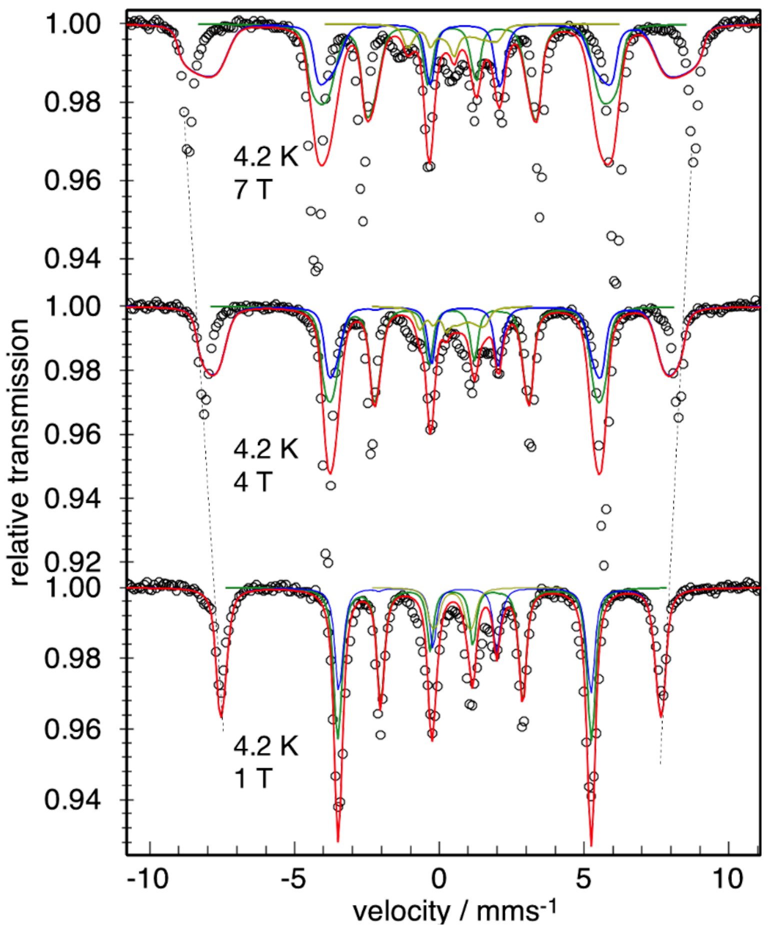

Figure S16. Magnetic Mössbauer spectra of a sample of **1** recorded at 4.2 K with fields of 1, 4 and 7 T applied perpendicular to the γ rays. The colored lines represent a global SH simulation by using the S2S1 model with the same electronic SH parameters as given in Figure 7 and with isomer shifts 0.65 and 0.47 mm/s, and quadrupole splittings 0.82 and 2.25 mm/s for Fe_{Td} and Fe_{SP} sites. Green: subspectrum of the Td site, blue: subspectrum of the SP site, dark yellow: 8% impurity ($S = 0$, $\delta = 0.45$ mm/S, $\Delta E_Q = 1.25$ mm/s), red: 1:1 superposition of subspectra. The dotted vertical lines are shown to emphasize the increase of the overall magnetic splitting with increasing applied fields, revealing the presence of a positive internal field.

The electric field gradient (*efg*) tensor of the SP site was rotated like the *D* and *g* matrices to keep its main component V_{zz} along the magnetic *z* axis (i.e. perpendicular to the ligand plane). In contrast, the *efg* of the Td site had to be slightly rotated out of the *z*-direction in order to achieve mainly the correct relative position and intensity of the left '$\Delta m_I = 0$' (line 2 of the 'green' subspectrum) due to suitable mixing of m_I substates for Fe_{Td}. The asymmetry parameter for both irons sites was kept at $\eta = 0$ (locally axis symmetry of the charge distributions). The main component V_{zz} (determining the sign of the quadrupole splitting) is positive for both sites.

The magnetic Mössbauer spectra provide a clear experimental assignment of isomer shifts and *efg* parameters to the Fe_{Td} and Fe_{SP} sites. The positive signs of the corresponding A_{zz} hyperfine coupling values are inferred from the field dependence of the spectra (Figure S16), which exhibit a significant increase of the overall magnetic splitting for both subspectra due to a positive sign of the internal field $B_{int} = -\langle S \rangle A / g_n\beta_n$, where $\langle S \rangle$ is the spin expectation value (negative for the lowest m_s level in the field). The trend of the spectra is reproduced by the corresponding spin Hamiltonian parameters, but we cannot reproduce the narrow experimental linewidths of these spectra. This may arise from torqueing of sample crystallites by the stronger fields. Therefore, we refrained from further refinement of the spin Hamiltonian description of the hydride dimer from the Mössbauer field dependence.

Considering the results of CASSCF studies (see below), we also modeled an isolated $S_{total} = 3$ system. This model can also provide a good global fit of magnetic susceptibility and Mössbauer data. In this model the nesting of the $M(\mu_B B/kT)$ curves did not fit quite as well (Figure S17); however note that *E/D* was fixed to 0 in the fit, whereas the CASSCF calculations predicted a large *E/D* of 0.29, which might explain the deviation. In this simulation, axial symmetry of the *g* matrix was assumed, because the magnetic data are not sensitive to $g_{t,y}$, and its value cannot be accurately determined. As shown in Figure S18, the low-lying doublet hardly splits when the applied field aligns along *y*.

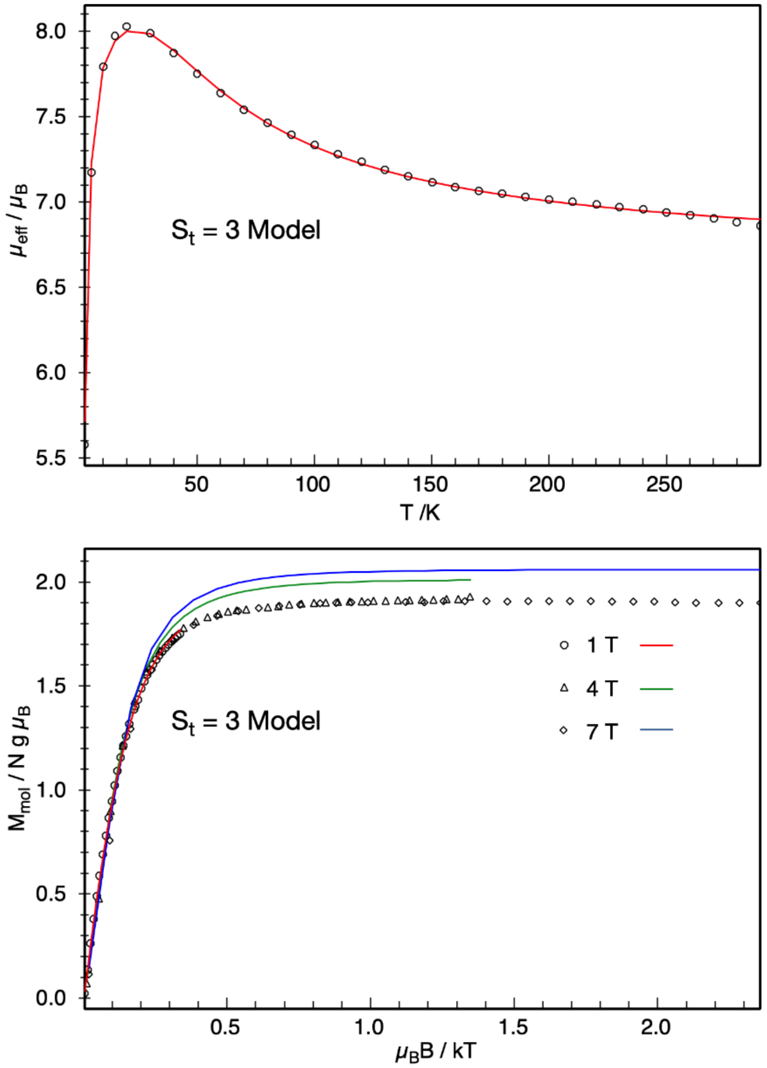

Figure S17. Top: Temperature dependence of the effective magnetic moment of **1** recorded with 1 T applied field. Bottom: 1/*T* dependence of the iso-field magnetization recorded with 1, 4 and 7 T. The experimental data were corrected for a TIP-like contribution to χ of 600 x 10^{-6} emu. The colored lines represent a global SH simulation using the **S_t = 3 model** and the following SH parameters: $g_t = (1.44, 1.44, 2.63)$, $D_t = -11.0\ \mathrm{cm}^{-1}, \frac{E}{D} = 0$.

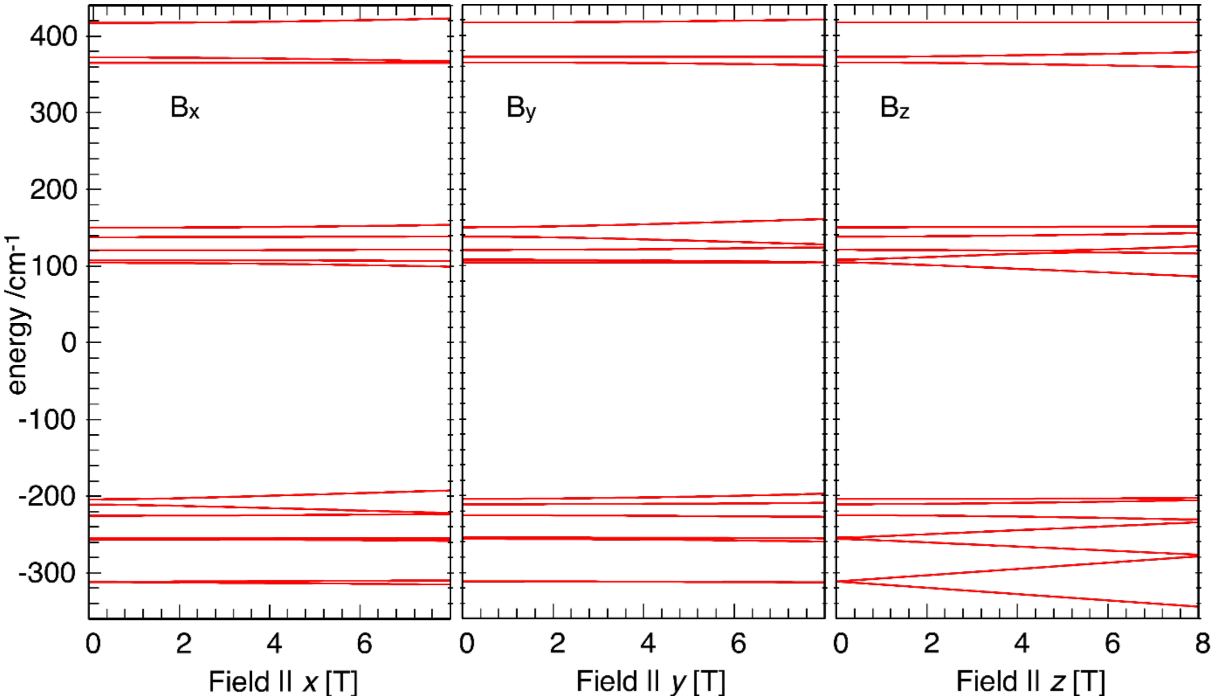

Figure S18. Energy level plot for the spin Hamiltonian simulation of the magnetic properties of **1** as used for Figures 7 and 8 using the S2S1 model with parameters with $S_{Td} = 2$, $S_{SP} = 1$ and the SH parameters: $J(\mathrm{Td} - \mathrm{SP}) = +63\ \mathrm{cm}^{-1}$, $g_{\mathrm{Td}} = (2.47, 2.47, 2.61)$, $g_{\mathrm{SP}} = (3.65, 3.65, 1.)$, $D_{Td} = -26.1\ \mathrm{cm}^{-1}, \frac{\mathrm{E}}{\mathrm{D}} = 0$, $D_{SP} = +40\ \mathrm{cm}^{-1}, \frac{\mathrm{E}}{\mathrm{D}} = 0$. The *D* and *g* matrices of the SP site are rotated with respect to the principal axes for the Td site by an Euler angle β =98°. The energies of the levels are shown as function of the applied field (B) in Tesla along the canonical *x,y,z* axes of the Td site. Note the anisotropic splitting of the ground state doublet in dependence of field direction.

Magnetic Mössbauer spectra were recorded on a conventional spectrometer with alternating constant acceleration of the γ-source (MPI-CEC, own construction). The sample temperature was maintained constant in a cryogen-free closed-cycle Mössbauer cryostat from Cryogenic Ltd, equipped with top-loading variable-temperature insert (VTI) and a split-pair superconducting magnet for fields up to 7 T, which were oriented perpendicular to the γ rays here. The ^{57}Co/Rh source (1.8 GBq) was kept at room temperature and was positioned inside the gap of the magnet system by using a re-entrant bore tube. The source was adjusted horizontally to a zero-field position in ca. 9 cm distance from the sample. The sample (KCM-II-121) was kept at 4.2 K. The minimum experimental line width was 0.3 mm/s (full width at half-height). The detector was a Si-Drift diode (150 mm^2 SDD CUBE) of a AXAS-M1 system from Ketek GmbH, mounted at the tip of a vacuum-tight 200 mm stainless steel finger. This finger was also inserted horizontally into the cryostat in the gap of the magnet to approach the sample to increase the aperture of the γ beam. The magnetic Mössbauer spectra were simulated with the program *mx.SL* (by E.B.) by diagonalization of the usual spin Hamiltonian.

Calculations. All calculations were performed using the ORCA quantum chemical program package.[20] Geometry optimizations were performed with BP86,[21] B3LYP, or TPSSh[22] density functional. The respective def2-TZVP (Fe, N, and the bridging H atoms) and def2-SVP basis sets[23] were applied in combination with the auxiliary basis sets def2/J.[24] The RI[25] and RIJCOSX [26] approximations were used to accelerate the calculations. The noncovalent interactions were taken into account *via* atom-pairwise dispersion corrections with Becke−Johnson (D3BJ) damping.[27]

The Mössbauer spectroscopic parameters were computed using the same density functional as for the geometry optimization step. We used the CP(PPP)[28] basis set for Fe, the TZVP[29] basis set for N, and the bridging H atoms and the SV(P) basis set[30] for remaining atoms.

Isomer shifts δ were calculated from the electron densities ρ_0 at the Fe nuclei by employing the linear regression:

$$\delta = \alpha \cdot (\rho_0 - C) + \beta \quad (8)$$

Here, C is a prefixed value, and α and β are the fit parameters. Their values for different combinations of the density functionals and basis sets can be found in our earlier work.[31]

Quadrupole splittings ΔE_Q were obtained from electric field gradients V_{ij} ($i = x, y, z$; V_{ii} are the eigenvalues of the electric field gradient tensor) by using a nuclear quadrupole moment $Q(^{57}Fe) = 0.16$ barn:[32]

$$\Delta E_Q = \frac{1}{2} eQ \cdot V_{zz} \cdot \sqrt{1 + \frac{1}{3}\eta^2} \quad (9)$$

Here, $\eta = \frac{V_{xx} - V_{yy}}{V_{zz}}$ is the asymmetry parameter.

For complete active space self-consistent field (CASSCF) computations,[33] the active space was chosen to distributed 14 electrons into 11 orbitals including all Fe 3*d* orbitals of the Td and SP sites, as well as the σ-bonding counterpart of the Fe_{Sp} d_{yz} orbital. Quasi-restricted orbitals derived from DFT calculations were used as the initial orbitals. To capture dynamic correction, on top of CASSCF wavefunctions, the second-order N-electron valence perturbation theory (NEVPT2)[34] was employed.

The geometry-optimized computational models at the BP86/def2-TZVP, B3LYP/def2-TZVP and TPSSh/def2-TZVP levels of theory unanimously demonstrated the lowest-energy nonet state of **1** to be higher in energy than the corresponding septet (by 29.3, 10.5 and 16.8 kcal/mol, respectively). Note that these differences exceed the ±7 kcal/mol uncertainty level that is estimated for spin-state energetics by DFT;[35] hence, these strongly support the S2S1 model of complex **1** with an $S = 3$ ground state. For both the S2S1 and S2S2 models, the dihedral angles between the two Fe-nacnac planes are roughly 90°, likely arising from steric repulsion between the bulky aryl groups. As elaborated below, the potential energy surfaces with respect to the dihedral angle (θ) between the equatorial plane of the Fe_{SP} site and the Fe_2H_2 core are rather flat, and the location of the energy minimum in the calculations is not robust. Because of this ambiguity, we also calculated the Mössbauer parameters for these geometries. As summarized in Table S4, the S2S1 model at the experimental geometry (from neutron crystallography) predicted Mössbauer parameters most accurately. Therefore, the geometry obtained from neutron diffraction was used for the more detailed information on the electronic structure.

Table S4. Comparison of computed Mössbauer parameters for the S2S1 and S2S2 models to the experimental values at 80 K for **1**.

Model	Geometry	δ_{Td} (mm/s)	$\lvert\Delta E_{Q,Td}\rvert$ (mm/s)	δ_{SP} (mm/s)	$\lvert\Delta E_{Q,SP}\rvert$ (mm/s)
Experimentally observed		0.65	0.82	0.47	2.25
S2S1	Exp.	0.57	1.29	0.45	1.55
S2S2	Exp.	0.49	4.51	0.40	1.89
S2S1	BP86	0.45	2.31	0.49	1.33
S2S1	B3LYP	0.60	1.33	0.48	1.53
S2S1	TPSSh	0.58	1.22	0.45	1.59
S2S2	BP86	0.41	4.59	0.53	1.05
S2S2	B3LYP	0.55	4.29	0.65	1.59
S2S2	TPSSh	0.64	1.80	0.64	1.88

Table S5. Low-lying excited states of **1** and induced orbital angular momentum of its ground state.

Root	CASSCF/NEVPT2 Excitation Energy (cm^{-1})	Assignment	Induced orbital angular momentum
1	390	Fe_{Td} $d_{xy} \longrightarrow d_{z2/x2\text{-}y2}$	L_z
2	770	Fe_{SP} $d_{x2-y2} \longrightarrow d_{xz}$	L_y
3	1250	Fe_{SP} $d_{x2\text{-}y2} \longrightarrow d_{xy}$	L_z

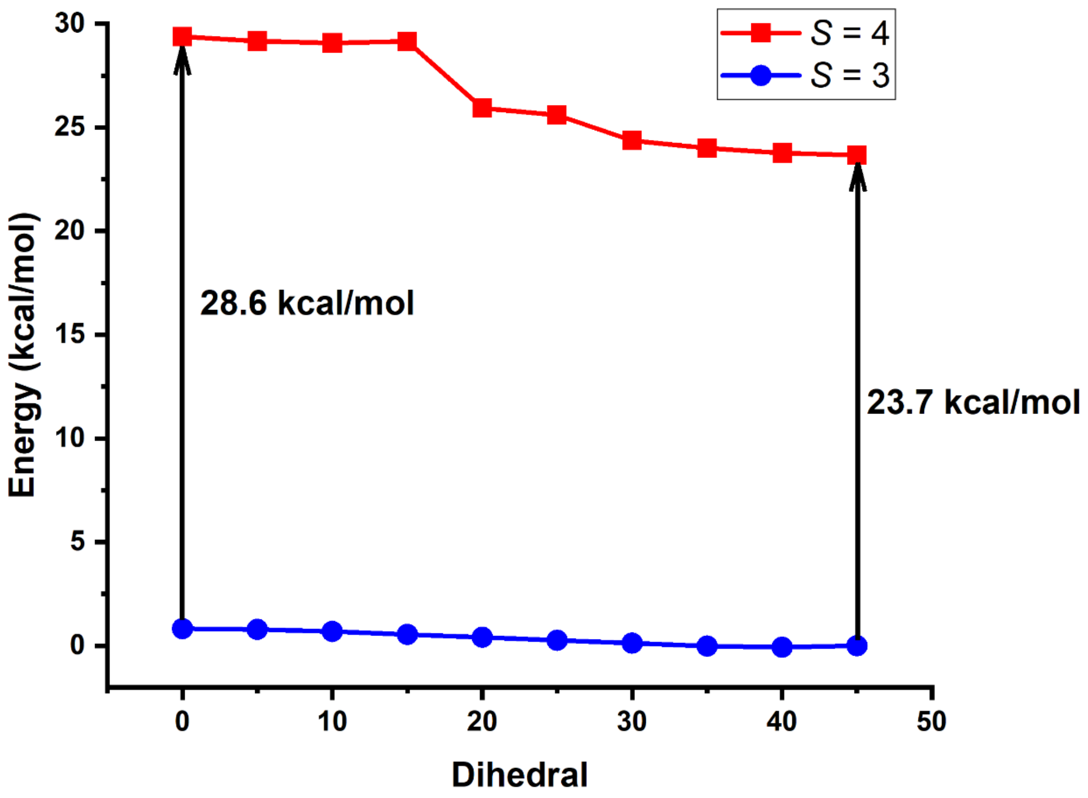

Figure S19. BP86-computed energy variations of the septet (blue) and nonet (red) states as a function of the dihedral angle between the $N_{Sp}Fe_{Sp}N_{Sp}$ and $HFe_{Sp}H$ planes. For the B3LYP energies, see the Figure 10 in the main text.

Example of the input file for CASSCF/NEVPT2 calculations

```
! def2-tzvp def2/jk rijk pal8

%basis newgto C "def2-SVP" end
        newgto H "def2-SVP" end
        end

%casscf
        nel 14
        norb 11
        nroots 10
        mult  7
        nevpt2 sc
        end
        rel
        dosoc true
        gtensor true      end
        end
*xyz 0 7
```

BP86-optimized geometry

Fe	9.987067	3.562900	11.393754
Fe	9.051893	5.200915	12.937576
H	9.268171	5.063462	11.295248
H	10.143961	3.949768	13.006855
N	8.244893	5.041587	14.644105
N	9.108052	7.108350	13.020351
C	7.253450	5.574683	16.819194
H	6.286093	6.094864	16.971453
C	7.923750	5.999684	15.526663
C	8.183136	7.378907	15.271448
C	8.720795	7.885906	14.053034
C	8.848065	9.389260	13.906507
H	9.236840	9.667842	12.911190
H	9.529985	9.811571	14.673598
H	7.869076	9.890792	14.050879
C	7.830629	8.400265	16.337839
H	7.525225	7.929080	17.287325
H	6.996565	9.069670	16.029659
H	8.686389	9.066420	16.577371
C	7.873941	3.675958	14.790656
C	6.626517	3.249657	14.258138
C	6.294620	1.885899	14.347904
H	5.327268	1.546251	13.946336
C	7.178596	0.964514	14.930273
H	6.906497	-0.100430	14.985974
C	8.411382	1.401408	15.441193
C	8.779538	2.758530	15.389022
C	5.712574	4.250553	13.600121
H	5.510464	5.120551	14.256519
H	4.750245	3.788870	13.311837
H	6.175618	4.657305	12.674731
C	10.097921	3.247773	15.920422
H	10.711432	3.680218	15.100620
H	9.967882	4.058126	16.666225
C	9.661478	7.665847	11.832025
C	11.057482	7.909110	11.769867
C	10.777001	8.629025	9.448174
C	9.398244	8.376143	9.527408
H	8.754079	8.552343	8.651253
C	8.817871	7.892623	10.714136
C	11.919075	7.598151	12.964314
H	11.602145	8.164424	13.863647
H	11.838188	6.522463	13.231656
C	7.353187	7.560515	10.808955

H	7.216454	6.467202	10.966717
H	6.811053	7.844186	9.886723
H	6.868165	8.059158	11.672094
N	11.725784	2.817058	11.136894
N	9.279037	2.728668	9.848395
C	11.283954	1.646060	9.032388
C	9.893053	1.950880	8.944455
C	9.054688	1.378847	7.816940
H	7.991823	1.656759	7.938294
H	9.117680	0.272316	7.781236
H	9.392430	1.754983	6.829055
C	11.917884	0.799081	7.943514
H	11.228607	0.610110	7.103334
C	11.727559	1.209238	13.497793
H	10.658849	1.519203	13.466784
H	11.898518	0.665173	14.446385
H	11.866499	0.503234	12.654451
C	7.873483	2.944490	9.886459
C	7.318556	4.083535	9.242333
C	5.936097	4.311185	9.370884
H	5.493820	5.192240	8.879674
C	5.122589	3.434206	10.106287
H	4.042404	3.626937	10.191759
C	5.687888	2.313271	10.733928
H	5.052665	1.625019	11.312886
C	7.066978	2.052329	10.644928
C	8.214137	5.020247	8.479966
H	8.958752	5.484404	9.162777
H	7.634952	5.829099	7.995991
H	8.799076	4.489947	7.701528
C	7.702754	0.881119	11.348183
H	8.329396	0.276423	10.662568
H	6.940275	0.225317	11.807286
H	8.373284	1.228085	12.164430
H	7.888328	5.806737	17.699470
H	7.059391	4.486619	16.820773
H	9.105902	0.678850	15.898073
H	10.674229	2.428565	16.390111
C	11.595806	8.395376	10.564188
H	12.679101	8.585495	10.502598
H	11.215378	9.006855	8.512024
H	12.983204	7.827642	12.766382
C	13.566722	1.560352	10.094594
H	14.110259	1.942273	9.205431
H	13.615908	0.453670	10.036648

H	14.113485	1.885699	10.997188
C	12.131148	2.046110	10.106792
H	12.819095	1.281632	7.508948
C	12.607477	3.186733	12.192353
C	12.623868	2.412484	13.381380
C	13.448332	2.833667	14.440351
H	13.469217	2.241905	15.369355
C	14.234709	3.991278	14.327266
H	14.873847	4.307594	15.165557
C	14.202459	4.745647	13.143927
H	14.812705	5.658406	13.055305
C	13.391275	4.361428	12.060293
C	13.301132	5.177369	10.798744
H	13.575639	4.586414	9.901168
H	13.959237	6.065652	10.843864
H	12.258973	5.525599	10.634323
H	12.249915	-0.195995	8.315322

B3LYP-optimized geometry

Fe	10.178841	3.654998	11.285939
Fe	9.044807	5.193866	12.933148
H	9.733867	5.330312	11.443638
H	9.469062	3.602921	12.897132
N	8.132974	4.996794	14.616953
N	9.087307	7.120088	13.083706
C	7.293657	5.528660	16.855015
H	6.435391	6.176957	17.087230
C	7.940258	5.929424	15.543477
C	8.276952	7.299361	15.366642
C	8.733508	7.850552	14.144639
C	8.813276	9.362297	14.030270
H	8.920921	9.678819	12.986933
H	9.682589	9.752546	14.585703
H	7.918819	9.842501	14.450931
C	8.056672	8.230925	16.546412
H	8.344142	7.758502	17.496915
H	7.003313	8.552427	16.662329
H	8.659544	9.144011	16.471342
C	7.665338	3.660390	14.798079
C	6.381283	3.319690	14.328172
C	5.962190	1.986232	14.432149
H	4.966103	1.713646	14.072387
C	6.796916	1.012559	14.981857
H	6.458853	-0.024402	15.051443
C	8.066831	1.367563	15.443668

C	8.518694	2.690480	15.366233
C	5.497171	4.376789	13.721818
H	5.323355	5.210192	14.420759
H	4.523671	3.959444	13.428164
H	5.963773	4.813104	12.824039
C	9.889664	3.082037	15.843035
H	10.490921	3.482970	15.010128
H	9.845760	3.878489	16.602456
C	9.529300	7.743966	11.877892
C	10.899634	8.025210	11.706613
C	10.425865	8.747033	9.429603
C	9.070945	8.466315	9.617532
H	8.360000	8.632863	8.803234
C	8.603666	7.961680	10.837296
C	11.872614	7.722477	12.812885
H	11.633105	8.272802	13.736354
H	11.840622	6.652132	13.073987
C	7.158262	7.596505	11.036976
H	7.051103	6.510178	11.200267
H	6.551681	7.867042	10.160670
H	6.728708	8.086018	11.924967
N	11.916539	2.786331	11.017023
N	9.421586	2.630723	9.789597
C	11.391813	1.437804	9.063695
C	10.012069	1.775668	8.965209
C	9.160516	1.137555	7.884307
H	8.112713	1.447089	7.980160
H	9.202511	0.039031	7.936062
H	9.509521	1.429225	6.881015
C	11.987309	0.476826	8.049493
H	11.295066	0.246939	7.233125
C	11.939813	1.250918	13.430631
H	10.869514	1.463013	13.271967
H	12.054505	0.810589	14.431257
H	12.222115	0.492175	12.683705
C	8.038969	2.950071	9.791705
C	7.594630	4.107238	9.112108
C	6.249701	4.478872	9.237583
H	5.896202	5.372400	8.716401
C	5.367221	3.732644	10.020508
H	4.322809	4.040722	10.112378
C	5.822990	2.597145	10.692932
H	5.138076	2.018204	11.316999
C	7.158932	2.190497	10.597561
C	8.560291	4.927123	8.298371

H	9.321221	5.397039	8.943366
H	8.040209	5.731573	7.758969
H	9.102265	4.309785	7.565197
C	7.667397	0.993148	11.354147
H	8.177225	0.276185	10.691874
H	6.849120	0.475514	11.871761
H	8.400682	1.297319	12.118700
H	8.005524	5.637096	17.689218
H	6.948604	4.489137	16.838493
H	8.723020	0.607117	15.876301
H	10.423805	2.222801	16.273342
C	11.329750	8.526010	10.471278
H	12.393154	8.736347	10.325618
H	10.778082	9.136157	8.471025
H	12.901463	7.975750	12.519108
C	13.688863	1.385058	10.090992
H	14.218830	1.667596	9.167410
H	13.704731	0.285139	10.135835
H	14.255350	1.776695	10.944036
C	12.269196	1.912037	10.079287
H	12.897751	0.882596	7.577671
C	12.766143	3.216422	12.070767
C	12.761330	2.504847	13.291998
C	13.510265	3.008545	14.362422
H	13.509330	2.466992	15.312294
C	14.245456	4.188584	14.231910
H	14.824215	4.569423	15.076978
C	14.234136	4.884285	13.021876
H	14.801320	5.813450	12.920822
C	13.493646	4.418266	11.927910
C	13.436951	5.188358	10.635045
H	13.700580	4.558486	9.771255
H	14.118927	6.050392	10.657051
H	12.419172	5.569896	10.447372
H	12.278106	-0.490394	8.499063

TPSSh-optimized geometry

Fe	10.115398	3.619318	11.300772
Fe	9.151704	5.233496	12.970801
H	9.913334	5.334973	11.500045
H	9.387598	3.592821	12.882315
N	8.205397	5.039933	14.612511
N	9.133017	7.136108	13.082519
C	7.211328	5.577396	16.790310
H	6.318782	6.195516	16.969601

C	7.911224	5.987182	15.509792
C	8.201191	7.360819	15.313667
C	8.715689	7.892776	14.106781
C	8.781411	9.400715	13.961927
H	8.950315	9.692138	12.918384
H	9.605667	9.818687	14.564265
H	7.851937	9.870368	14.314279
C	7.871439	8.327645	16.436599
H	7.973037	7.852481	17.421748
H	6.840165	8.726113	16.378353
H	8.548704	9.192911	16.442355
C	7.773858	3.690949	14.811315
C	6.503238	3.306166	14.336156
C	6.121719	1.963403	14.465547
H	5.138215	1.653711	14.101615
C	6.980114	1.025651	15.044591
H	6.671129	-0.018527	15.133328
C	8.235521	1.426715	15.511802
C	8.649091	2.761146	15.411078
C	5.605382	4.328587	13.693061
H	5.385586	5.161951	14.379018
H	4.655897	3.877572	13.373601
H	6.092358	4.768655	12.807282
C	10.004412	3.211370	15.879546
H	10.575918	3.637446	15.037214
H	9.929767	4.004757	16.639790
C	9.642231	7.750316	11.895379
C	11.015255	8.059717	11.814302
C	10.668068	8.791703	9.516986
C	9.309856	8.477698	9.615844
H	8.648439	8.634572	8.759196
C	8.776853	7.953259	10.800802
C	11.916832	7.769236	12.982747
H	11.621731	8.334381	13.880689
H	11.860036	6.701243	13.251383
C	7.330212	7.558769	10.914826
H	7.243196	6.485738	11.158922
H	6.791685	7.743060	9.974395
H	6.819705	8.103969	11.724454
N	11.838920	2.776339	10.999648
N	9.360780	2.632706	9.811200
C	11.329046	1.485058	9.009789
C	9.945879	1.803062	8.946127
C	9.079752	1.183386	7.867200
H	8.030494	1.476209	8.000305

H	9.139216	0.084493	7.887527
H	9.401594	1.511325	6.865861
C	11.923721	0.565790	7.959551
H	11.219584	0.361341	7.145402
C	11.838332	1.190411	13.372508
H	10.769443	1.439254	13.256979
H	11.973807	0.705812	14.349625
H	12.081728	0.461405	12.584016
C	7.966781	2.905259	9.845366
C	7.468996	4.058196	9.194678
C	6.103492	4.350943	9.315068
H	5.705966	5.237580	8.814316
C	5.254744	3.531236	10.062991
H	4.193294	3.775502	10.146716
C	5.766240	2.404237	10.711934
H	5.106821	1.768837	11.308168
C	7.124564	2.076525	10.624488
C	8.398748	4.943944	8.408309
H	9.175581	5.375089	9.061144
H	7.848962	5.773241	7.941571
H	8.921532	4.381755	7.618672
C	7.696373	0.890074	11.352408
H	8.244088	0.219523	10.672064
H	6.904045	0.317380	11.852397
H	8.410849	1.218451	12.125410
H	7.877679	5.712621	17.658232
H	6.907620	4.524445	16.759012
H	8.909029	0.694973	15.966527
H	10.578535	2.375463	16.303672
C	11.510931	8.580413	10.611644
H	12.576232	8.815259	10.534524
H	11.070422	9.197322	8.585810
H	12.961878	8.015767	12.747153
C	13.637858	1.441637	10.016267
H	14.158777	1.779931	9.106004
H	13.674193	0.341221	10.011647
H	14.192565	1.808682	10.888915
C	12.209125	1.939723	10.030081
H	12.824674	1.000738	7.495535
C	12.687070	3.181817	12.065172
C	12.683438	2.431970	13.264653
C	13.453879	2.891563	14.340356
H	13.458203	2.320481	15.272535
C	14.203207	4.066501	14.236805
H	14.798148	4.412359	15.085140

C	14.184689	4.802592	13.049411
H	14.762612	5.727119	12.971115
C	13.425693	4.381365	11.949132
C	13.366234	5.185636	10.677620
H	13.692465	4.594701	9.807292
H	14.000654	6.080164	10.748806
H	12.334040	5.514499	10.472276
H	12.226293	-0.412116	8.376530

Note on literature diketiminate-supported iron-hydride complexes

It is worth reflecting on previously reported dimeric iron(II) hydride complexes supported by bulkier diketiminate ligands, which gave only one doublet in Mössbauer spectra. For example, with the $L^{tBu,iPr}$ ligand, the observed Mössbauer parameters at 80 K were $\delta = 0.58$ mm/s and $|\Delta E_Q| = 1.74$ mm/s, and with the $L^{Me,iPr}$ ligand, $\delta = 0.51$ mm/s and $|\Delta E_Q| = 2.05$ mm/s.[36] The isomer shifts were near the low end of the range observed for diketiminate-supported (three-coordinate) high-spin iron(II) alkyl and aryl complexes (e.g. $L^{tBu,iPr}FeCH_3$ at $\delta = 0.48$ mm/s),[37] and thus were not anomalous enough to raise concerns. However, in retrospect, it is notable that these values fall between the parameters for the two *different* but interconverting sites in **1**. Thus, it is possible that the earlier hydride compounds have analogous dynamic behavior, but we observed an average because they did not fortuitously crystallize in an environment that differentiated the sites.

References

[1] Rodriguez, M. M.; Bill, E.; Brennessel, W. W.; Holland, P. L. *Science* **2011,** *334*, 780.

[2] Pelmenschikov, V.; Gee, L. B.; Wang, H.; MacLeod, K. C.; McWilliams, S. F.; Skubi, K. L.; Cramer, S. P.; Holland, P. L. *Angew. Chem. Int. Ed.* **2018,** *57*, 9367. See also: Dugan, T. R.; Bill, E.; MacLeod, K. C.; Brennessel, W. W.; Holland, P. L. *Inorg. Chem.* **2014,** *53*, 2370.

[3] Blume, M.; Tjon, J. A. *Phys. Rev.* **1968,** *165*, 446.

[4] Litterst, F. J.; Amthauer, G. *Physics and Chemistry of Minerals* **1984,** *10*, 250.

[5] Wickman, H. H.; Klein, M. P.; Shirley, D. A. *Phys. Rev.* **1966,** *152*, 345.

[6] Li, J.; Noll, B. C.; Oliver, A. G.; Schulz, C. E.; Scheidt, W. R. *J. Am. Chem. Soc.* **2013,** *135*, 15627.

[7] Carver, G.; Dobe, C.; Jensen, T. B.; Tregenna-Piggott, P. L. W.; Janssen, S.; Bill, E.; McIntyre, G. J.; Barra, A. L. *Inorg. Chem.* **2006,** *45*, 4695.

[8] Kahn, O. *Molecular Magnetism*; VCH: New York, 1993.

[9] Sorai, M.; Seki, S. *J. Phys. Chem. Solids* **1974**, *35*, 555.

[10] Bruker, *APEX2*, *SADABS*, Bruker AXS Inc., Madison, Wisconsin, USA, 2014.

[11] Sheldrick, G. M. *Acta Cryst. C* **2015,** *71*, 3.

[12] Coates, L.; Cao, H. B.; Chakoumakos, B. C.; Frontzek, M. D.; Hoffmann, C.; Kovalevsky, A. Y.; Liu, Y.; Meilleur, F.; dos Santos, A. M.; Myles, D. A. A.; Wang, X. P.; Ye, F., *Rev. Sci Instrum.* **2018,** *89*, 092802.

[13] Schultz, A. J.; Jørgensen, M. R. V.; Wang, X.; Mikkelson, R. L.; Mikkelson, D. J.; Lynch, V. E.; Peterson, P. F.; Green, M. L.; Hoffmann, C. M. *J. Applied Crystallogr.* **2014,** *47* (3), 915-921.

[14] Zikovsky, J.; Peterson, P. F.; Wang, X. P. P.; Frost, M.; Hoffmann, C. *J. Applied Crystallogr.* **2011,** *44*, 418.

[15] Henning, R. W.; Schultz, A. J.; Hitchman, M. A.; Kelly, G.; Astley, T. *Inorg. Chem.* **2000,** *39*, 765.

[16] Bencini, A.; Gatteschi, D., *EPR of Exchange Coupled Systems*. Springer Verlag: Berlin, 1990.

[17] Keilwerth, M.; Hohenberger, J.; Heinemann, F. W.; Sutter, J.; Scheurer, A.; Fang, H.; Bill, E.; Neese, F.; Ye, S.; Meyer, K. *J. Am. Chem. Soc.* **2019,** *141*, 17217-17235.

[18] Cheng, J.; Liu, J.; Leng, X.; Lohmiller, T.; Schnegg, A.; Bill, E.; Ye, S.; Deng, L. *Inorg. Chem.* **2019,** *58*, 7634-7644.

[19] Tarrago, M.; Römelt, C.; Nehrkorn, J.; Schnegg, A.; Neese, F.; Bill, E.; Ye, S. *Inorg. Chem.* **2021,** *60*, 4966-4985.

[20] Neese, F. *WIREs Comput. Mol. Sci.* **2012**, *2*, 73.

[21] (a) Becke, A. D. *Phys. Rev. A* **1988**, *38*, 3098. (b) Perdew, J. P. *Phys. Rev. B* **1986**, *33*, 8822.

[22] Staroverov, V. N.; Scuseria, G. E.; Tao. J.; Perdew, J. P. *J. Chem. Phys.* **2004**, *121*, 11507.

[23] Weigend, F.; Ahlrichs, R. *Phys. Chem. Chem. Phys.* **2005**, *7*, 3297.

[24] F. Weigend, *J. Comput. Chem.* **2008,** *29*, 167.

[25] Eichkorn, K.; Treutler, O.; Öhm, H.; Häser, M.; Ahlrichs, R. *Chem. Phys. Lett.* **1995**, *242*, 652.

[26] Neese, F.; Wennmohs, F.; Hansen, A.; Becker, U. *Chem. Phys.* **2009**, 356, 98.

[27] Grimme, S.; Ehrlich, S.; Goerigk, L. *J. Comput. Chem.* **2011,** *32*, 1456.

[28] Neese, F. *Inorg. Chim. Acta* **2002**, *337*, 181.

[29] Schäfer, A.; Huber, C.; Ahlrichs, R. *J. Chem. Phys.* **1994**, *100*, 5829.

[30] Moore, J. T.; Chatterjee, S.; Tarrago, M.; Clouston, L. J.; Sproules, S.; Bill, E.; Bernales, V.; Gagliardi, L.; Ye, S.; Lancaster, K. M.; Lu, C. C. *Inorg. Chem.* **2019**, *58*, 6199.

[31] Römelt, M.; Ye, S.; Neese, F. *Inorg. Chem.* **2009**, *48*, 784.

[32] Sinnecker, S.; Slep, L. D.; Bill, E.; Neese, F. *Inorg. Chem.* **2005,** *44*, 2245.

[33] (a) Roos, B. O. *Adv. Chem. Phys.* **2007**, *69*, 399. (b) Ruedenberg, K.; Cheung, L. M.; Elbert, S. T. *Int. J. Quantum Chem.* **1979**, *16*, 1069−1101. (c) Roos, B. O.; Taylor, P. R.; Siegbahn, P. E. M. *Chem. Phys.* **1980**, *48*, 157.

[34] (a) Angeli, C.; Borini, S.; Cestari, M.; Cimiraglia, R. *J. Chem. Phys.* **2004**, *121*, 40434049. (b) Angeli, C.; Cimiraglia, R.; Malrieu, J.-P. *J. Chem. Phys.* **2002**, *117*, 9138.

[35] Ye, S.; Neese, F. *Inorganic Chemistry* **2010,** *49*, 772-774.

[36] Dugan, T. R.; Bill, E.; MacLeod, K. C.; Brennessel, W. W.; Holland, P. L. *Inorg. Chem.* **2014,** *53*, 2370-2380.

[37] Andres, H.; Bominaar, E.; Smith, J. M.; Eckert, N. A.; Holland, P. L.; Münck, E. *J. Am. Chem. Soc.* **2002,** *124*, 3012-3025.
